# Structure of the space of taboo-free sequences

**DOI:** 10.1007/s00285-020-01535-5

**Published:** 2020-09-17

**Authors:** Cassius Manuel, Arndt von Haeseler

**Affiliations:** 1 Center for Integrative Bioinformatics Vienna, Max Perutz Labs, University of Vienna, Medical University of Vienna, Dr. Bohr Gasse 9, 1030 Vienna, Austria; 2 Faculty of Computer Science, University of Vienna, Währinger Str. 29, 1090 Vienna, Austria

**Keywords:** Bacteriophage DNA evolution, Endonuclease-dependent evolution, Restriction-enzyme dependent evolution, Restriction–modification system, Hamming graph with taboos, Connectivity of Hamming graphs, 05C40, 92D15

## Abstract

Models of sequence evolution typically assume that all sequences are possible. However, restriction enzymes that cut DNA at specific recognition sites provide an example where carrying a recognition site can be lethal. Motivated by this observation, we studied the set of strings over a finite alphabet with **taboos**, that is, with prohibited substrings. The taboo-set is referred to as $\mathbb {T}$ and any allowed string as a taboo-free string. We consider the so-called Hamming graph $\varGamma _n(\mathbb {T})$, whose vertices are taboo-free strings of length *n* and whose edges connect two taboo-free strings if their Hamming distance equals one. Any (random) walk on this graph describes the evolution of a DNA sequence that avoids taboos. We describe the construction of the vertex set of $\varGamma _n(\mathbb {T})$. Then we state conditions under which $\varGamma _n(\mathbb {T})$ and its suffix subgraphs are connected. Moreover, we provide an algorithm that determines if all these graphs are connected for an arbitrary $\mathbb {T}$. As an application of the algorithm, we show that about $87\%$ of bacteria listed in REBASE have a taboo-set that induces connected taboo-free Hamming graphs, because they have less than four type II restriction enzymes. On the other hand, four properly chosen taboos are enough to disconnect one suffix subgraph, and consequently connectivity of taboo-free Hamming graphs could change depending on the composition of restriction sites.

## Introduction

In bacteria, restriction enzymes cleave foreign DNA to stop its propagation. To do so, a double-stranded cut is induced by a so-called recognition site, a DNA sequence of length 4–8 base pairs (Alberts et al. 2004). As part of their restriction–modification (R–M) system, bacteria can escape the lethal effect of their own restriction enzymes by modifying recognition sites in their own DNA (Kommireddy and Nagaraja 2013). Nevertheless, Gelfand and Koonin (1997) and Rocha et al. (2001) found a significant avoidance of recognition sites in bacterial DNA, and Rusinov et al. (2015) showed that this avoidance was characteristic of type II R–M systems. Also in bacteriophages, the avoidance of the recognition sites is evolutionary advantageous (Rocha et al. 2001), mainly for non-temperate bacteriophages affected by orthodox type II R–M systems (Rusinov et al. 2018a). Therefore in those instances the recognition site is, as we call it, a **taboo** for host and foreign DNA.

Although avoidance of recognition sites is well studied, e.g. by Rusinov et al. (2018b), taboo free DNA evolution has not yet been modelled. To initiate models of sequence evolution with taboos, we studied the Hamming graph $\varGamma _n(\mathbb {T})$, whose vertices are strings of length *n* over a finite alphabet $\varSigma$ not containing any taboos of the set $\mathbb {T}$ as subsequence. Two vertices of the Hamming graph are adjacent if the corresponding taboo-free strings have Hamming distance equal to one. In biological terms, the sequences differ by a single substitution.

We note that, for a binary alphabet $\varSigma = \{ 0, 1 \}$ and taboo-set $\mathbb {T} = \{ 11 \}$, the corresponding Hamming graphs $\varGamma _n(\mathbb {T})$ are known as Fibonacci cubes. Some properties of the Fibonacci cubes like the Wiener Index or the degree distribution were surveyed by Klavžar (2013). Further results have been obtained for taboo-sets forbidding arbitrary numbers of consecutive “1”s, $\mathbb {T} = \{ 1 \ldots 1 \}$, by Hsu and Chung (1993), or when $\mathbb {T} = \{ s \}$ for an arbitrary binary string *s* by Ilić et al. (2012). Recently, the equivalent problem of lattice paths that avoid some patterns has been described using automata and generating functions by Asinowski et al. (2018, (2020).

We are not so much interested in enumerative properties of Hamming graphs. We want to define conditions under which the Hamming graphs stay connected for arbitrary finite alphabets and arbitrary finite taboo-sets. From an evolutionary point of view, connectivity guarantees that any taboo-free sequence can be generated by point mutations from any initial taboo-free sequence without containing a taboo-string during evolution. To include further biological realism, we will also study the connectivity of subgraphs $\varGamma _n^s(\mathbb {T})$ of the Hamming graph, where *s* is a taboo-free suffix. Suffix *s* can be viewed as a conserved DNA fragment, that is, a sequence that remained invariable during evolution (Shoemaker and Fitch 1989; Fitch and Margoliash 1967).

The inclusion of Hamming graphs with a constant suffix provides more general results, because ${\varGamma ^e_n(\mathbb {T}) = \varGamma _n(\mathbb {T})}$, where *e* is the empty string. Given a taboo-set $\mathbb {T}$, if for every taboo-free string *s* and integer *n* the Hamming graph $\varGamma _n^s(\mathbb {T})$ is connected, then evolution can explore the space of taboo-free sequences by simple point mutation, no matter which DNA suffix fragments remain invariable, as long as the taboo-set $\mathbb {T}$ does not change in the course of evolution.

## Motivating examples and non-technical presentation of key results

Here, we give a non-technical description of the essential results to determine connectivity. The subsequent sections provide a more technical and precise description of the central results.

**Fig. 1:**
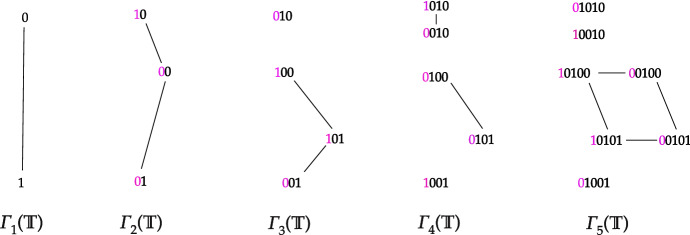
Graph $\varGamma _n(\mathbb {T})$ for $n \in [1, 5]$ for binary alphabet $\varSigma = \{ 0, 1\}$ and $\mathbb {T} = \{ 11,\; 000\}$. Set $V_{n+1}(\mathbb {T})$ is constructed by adding every allowed letter at the beginning of each string in $V_{n}(\mathbb {T})$

Consider an alphabet $\varSigma$, for example $\varSigma = \{ 0, 1\}$. In a **Hamming graph of length**  *n*, all possible words of length *n* are vertices, and two of these vertices are joined by an edge if they differ in exactly one position. A taboo-set is a set of forbidden subwords, such as $\mathbb {T} = \{ 11, 000\}$. Then, to construct a **taboo-free Hamming graph** $\varGamma _n(\mathbb {T})$, we simply have to erase all words of the Hamming graph of length *n* containing those taboos. Figure 1 provides an example where $\varGamma _n(\mathbb {T})$ is disconnected for $n \ge 3$.

**Fig. 2:**
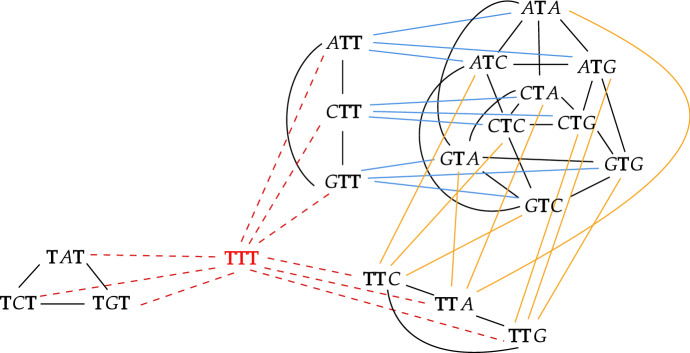
Graph $\varGamma _3(\mathbb {T})$, where $\varSigma = \{ A,C,G,T\}$ and ${\mathbb {T} = \{ AA, AC, AG, CA, CC,CG, GA,GC,GG\}.}$ Vertex *TTT* is a cut vertex, because if we remove *TTT* and its incident edges (dashed lines, coloured red), then the resulting graph is disconnected. Consequently, graph $\varGamma _3(\mathbb {T}^*)$ induced by taboo-set $\mathbb {T}^* = \mathbb {T} \bigcup \{ TTT \}$ is disconnected. Red, blue and yellow edges connect vertices with a different distribution of letter *T* (colour figure online)

Given some alphabet and some taboo-set, deciding whether graph $\varGamma _n(\mathbb {T})$ is connected is not a trivial task. To see this, consider the four-nucleotide alphabet $\varSigma = \{ A,C,G,T\}$, which is our main object of interest. Figure 2 shows the connected graph $\varGamma _3(\mathbb {T})$ for taboo-set ${\mathbb {T} = \{ AA, AC, AG, CA, CC,CG, GA,GC,GG\}}$. The word *TTT* is a cut vertex, meaning that taboo-set ${\mathbb {T}^* = \mathbb {T} \bigcup \{ TTT \}}$ yields the disconnected graph $\varGamma _3(\mathbb {T}^*).$

Since the addition or deletion of one single taboo can have such an impact on connectivity, we need a tool to determine the structure of the taboo-free Hamming graphs. This tool is described in full generality at the end of Sect. 8. In the particular case when $\varSigma = \{ A,C,G,T\}$, our results can be simplified as follows.

1. If the number of taboos is smaller than the size of the alphabet, that is if ${|\mathbb {T}| < 4}$, then all graphs $\varGamma ^s_n(\mathbb {T})$ are connected (Corollary 25.b). For example, given ${\mathbb {T} = \{ AATT, CCGG\}}$, all taboo-free Hamming graphs are connected.
  Similarly, if the size of the set of all starting letters of taboos is smaller than the size of the alphabet, then all taboo-free Hamming graphs are connected (Corollary 25.a). This applies for taboo-set ${\mathbb {T} \!=\! \{ AA, AC\!, AG, CA, CC\!,CG, GA,GC\!,GG\}}$, because the set of initial letters is ${\{ A,C,G\}}$ and ${|\{ A,C,G\}| = 3 < 4}$.
2. Proposition 24 describes a slightly more complex sufficient condition to determine connectivity. Given $\mathbb {T}$, delete the first letter of each taboo to construct the set $\varPsi (\mathbb {T})$. For example, if $\mathbb {T} = \{ AAA, CCA, GGA, TTT\}$, then ${\varPsi (\mathbb {T}) = \{ AA, CA, GA, TT\}}$.
  In set $\varPsi (\mathbb {T})$, consider every pair of strings with Hamming distances 1 or 0. For example, the pair (*AA*, *AA*) has distance 0; the pair (*AA*, *CA*) has distance 1; and the pair (*AA*, *TT*) has distance 2. If every pair with Hamming distance 1 or 0 can be taboo-free extended to the left by the same letter, then all graphs $\varGamma ^s_n(\mathbb {T})$ are connected.
  For example, the pair (*AA*, *AA*) can be extended by *C*, because *CAA* is taboo-free, and the pair (*AA*, *CA*) can be extended by *T*, because *TAA* and *TCA* are taboo-free. After checking all possible pairs with Hamming distance 0 or 1, we see that all such pairs in $\varPsi (\mathbb {T})$ are extendable to the left, and thus taboo-set $\mathbb {T}$ generates connected taboo-free Hamming graphs.
3. If Proposition 24 cannot be applied, then we apply the characterization of Theorem 22. Assume for example that $\mathbb {T} = \{ AAA, CCA, TAA, GAA\}$. Since the pair $\{AA, CA\} \subset \varPsi (\mathbb {T})$ with Hamming distance one is not taboo-free extendable to the left by any letter, we proceed as follows. First we construct ${{\,\mathrm{suf}\,}}(\mathbb {T})$, the set of all proper suffixes of $\mathbb {T}$. In our example, ${{\,\mathrm{suf}\,}}(\mathbb {T}) = \{ AA, CA, A, e\}$, where *e* is the string with no letters. Now we consider, for every suffix $r \in {{\,\mathrm{suf}\,}}(\mathbb {T})$ the graph $\varGamma ^r_{|r| + M}(\mathbb {T})$, where |*r*| is the length of *r* and *M* is the length of the longest taboo(s) in $\mathbb {T}$. If all graphs $\varGamma ^r_{|r| + M}(\mathbb {T})$ are connected, then every graph $\varGamma ^s_n(\mathbb {T})$ is connected. In our example, graphs $\varGamma ^{AA}_{5}(\mathbb {T})$, $\varGamma ^{CA}_{5}(\mathbb {T})$, $\varGamma ^{A}_{4}(\mathbb {T})$ and $\varGamma _{3}(\mathbb {T})$ are connected, implying that all taboo-free Hamming graphs are connected.
  When graph $\varGamma ^r_{|r| + M}(\mathbb {T})$ is disconnected for some $r \in {{\,\mathrm{suf}\,}}(\mathbb {T})$, then suffix *r* induces disconnected taboo-free Hamming graphs of the form $\varGamma ^r_n(\mathbb {T})$ for $n \ge |r| + M$. Therefore evolution cannot explore the whole space of taboo-free sequences. This is the case for taboo-set $\mathbb {T}^*$ of Fig. 2, where $r = e$ yields the disconnected graph $\varGamma _3(\mathbb {T}^*)$.

## Outline

We will characterize taboo-sets $\mathbb {T}$ such that every Hamming graph of the form $\varGamma ^s_n(\mathbb {T})$ is connected. To this end, we describe in Sect. 5 basic properties of taboo-sets. In Sect. 6, we introduce a very general type of taboo-sets, called **left proper** (Definition 4), which are our main object of study. In Proposition 11.b we show that, to construct graph $\varGamma _n^s(\mathbb {T})$, we only need the longest prefix of *s* which is a suffix of a taboo, which we call $s[1, k_s]$. In Sect. 7 we state the graph isomorphism ${\varGamma _n^s(\mathbb {T}) \simeq \varGamma ^{s[1, k_s]}_n(\mathbb {T})}$ (Theorem 16). In Sect. 8 we explain how the edges of a quotient graph are related to the structure of graph $\varGamma _n^n(\mathbb {T})$ (Proposition 17).

Combining all these results, in Sect. 8 we characterize the connectivity of Hamming graphs $\varGamma ^s_n(\mathbb {T})$. We prove by induction that the connectivity of a small number of quotient graphs implies the connectivity of all Hamming graphs with long suffixes (Proposition 20). This result can be used to prove connectivity of Hamming graphs with short suffixes (Proposition 21). These two results yield the characterization of the connectivity of every suffix Hamming graph in Theorem 22. Section 9 provides examples of bacterial taboo-sets and their connectivity.

## Basic notations

We will introduce some standard notations concerning strings as well as some relevant terms from graph theory.

### Strings

We will use the term **string** to refer to a sequence of symbols over an arbitrary finite alphabet $\varSigma = \{ a_1, \dots , a_m \}$, where $m \ge 2$, while **(DNA) sequence** is reserved for biological contexts, where the alphabet consists of the four nucleotides ${\varSigma = \{ A,C,G,T \}}$.

We denote the set of strings of length *n* over the alphabet $\varSigma$ by $\varSigma ^n$. The length of a string *s* is denoted by |*s*|. The empty string will be denoted by *e*, and satisfies $|e| = 0$ and $\{ e \} = \varSigma ^0$.

Given a string $s= b_1 \dots b_n \in \varSigma ^n$, the expression

$$\begin{aligned} s[i,j] := {\left\{ \begin{array}{ll} b_{i} \dots b_j &{}\text { if } 1 \le i \le j \le n\\ e &{}\text { otherwise } \end{array}\right. } \end{aligned}$$

denotes the **substring** of *s* starting at the *i*th position and ending at the *j*th position, and *e* when this substring is not well-defined (for example if $j = 0$). In particular *s*[1, *j*] is a **prefix** of *s* that ends at position *j* and *s*[*i*, *n*] is a **suffix** of *s* that starts at position *i*. A substring, prefix or suffix is called **proper** if it is not the entire string *s*. For a set of strings *S*, we define **the substrings from the** *i* **th to the** *j* **th position of** *S* as

$$\begin{aligned} S[i,j] := \{ s[i,j] \; | \; s\in S \}. \end{aligned}$$

We also need **the set of proper suffixes of** *S*, defined as

$$\begin{aligned} {{\,\mathrm{suf}\,}}(S) := \left( \bigcup _{s \in S} \bigcup _{i \in [2, |s|]} s[i, |s|] \right) \bigcup \{ e \}. \end{aligned}$$

where $i \in [2, |s|]$ refers to all integers *i* within the interval [2, |*s*|]. It should not be confused with substring *s*[2, |*s*|] of *s*.

#### Example 1

If $$S = \{ ACG,\; GGG,\; TTC,\; CC \}$$ then

$$\begin{aligned} {{\,\mathrm{suf}\,}}(S) =\{ CG,\; G,\; GG,\; TC,\; C,\; e \} . \end{aligned}$$

If string $$s_1$$ is substring of string $$s_2$$, we write $$s_1 \prec s_2$$, while $$s_1 \not \prec s_2$$ denotes that $$s_1$$ is **not** a substring of $$s_2$$. By convention, $$e \prec s$$ for any string *s*. For strings $$s_1$$ and $$s_2$$, we define $$s_1 s_2 $$ as the **concatenation** of $$s_1$$ and $$s_2$$. Note that $$e s = se = s$$ for any *s*. For a string *s* and a set of strings $${S = \{ s_1, \dots s_k \}}$$, the concatenation of *s* with all elements in *S* is denoted by $$s \circ S := \{ s s_1, \dots s s_k \}$$. If $$S_1$$ and $$S_2$$ are disjoint sets, then the disjoint union of $$S_1$$ and $$S_2$$ will be denoted by $$S_1 \bigsqcup S_2$$.

Finally, given two strings $s_1, s_2$ of equal length, $d(s_1, s_2)$ denotes their **Hamming distance**, that is, the number of positions at which the corresponding symbols differ.

### Graph theory

We will use common graph theory terminology following Wilson (1986). Let $\mathcal{G} =(V,E)$ denote a simple, undirected graph with vertex set *V* and edge set *E*. We say that graph $\mathcal{G}_1 = (V_1, E_1)$ is **subgraph** of $\mathcal{G}_2= (V_2, E_2)$ if $V_1 \subseteq V_2$ and $E_1 \subseteq E_2$, and we denote this as $\mathcal{G}_1 \subseteq \mathcal{G}_2$.

Given a graph $\mathcal{G} = (V,E)$ and a subset $V_1 \subseteq V$, then the **subgraph induced by** $V_1$ **in**  $\mathcal{G}$, $\mathcal{G}(V_1) = (V_1, E_{V_1})$, has vertex set $V_1$ and, for any $u, v \in V_1$, $\{ u, v\} \in E_{V_1}$ iff $\{ u, v\} \in E$.

Two graphs $\mathcal{G}_1 = (V_1, E_1)$ and $\mathcal{G}_2 = (V_2, E_2)$ are **isomorphic**, denoted by $\mathcal{G}_1 \simeq \mathcal{G}_2$, if there exists a bijection $f: V_1 \rightarrow V_2$ such that, for every $u, v \in V_1$, $\{u, v\} \in E_1$ iff $\{f(u), f(v)\} \in E_2$. That is, $\mathcal{G}_1$ and $\mathcal{G}_2$ are isomorphic if there exists an edge-preserving bijection between their vertex sets.

**Fig. 3:**
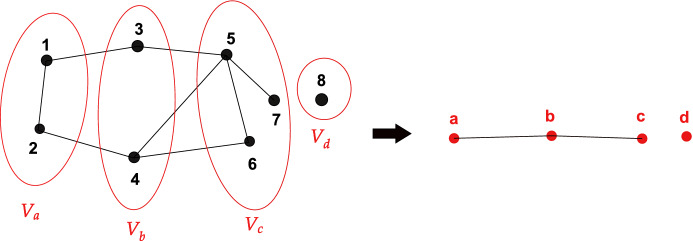
Example of a quotient graph. For $\mathcal{G} = (V, E)$ on the left hand side, with $V= \{ 1,2,3,4,5,6,7,8\}$ and partition $V = V_a \bigsqcup V_b \bigsqcup V_c \bigsqcup V_d$, we obtain the quotient graph $\mathcal{Q}[\mathcal{G}]$ on the right hand side

We will also need the **quotient graph**, as defined by Sanders and Schulz (2013), to study the connectivity of Hamming graphs. To define it, consider a graph $\mathcal{G} = (V,E)$ and a partition of its vertex set *V*, namely $V = \bigsqcup _{b \in J} V_b$ for some index set *J*. The **quotient graph of** $\mathcal{G}$, denoted as $\mathcal{Q}[\mathcal{G}] = (J, E_J)$, is the graph whose vertices are *J* and such that $\{ b_1, b_2 \} \in E_J$ iff an edge connects a vertex in $V_{b_1}$ with a vertex in $V_{b_2}$. Figure 3 gives an example of a quotient graph.

Our strategy to prove connectivity of taboo-free Hamming graphs will use the following propositions, whose proof is simple enough to be omitted.

#### Proposition 1

Consider graph $\mathcal{G} = (V,E)$ and partition $V = \bigsqcup _{b \in J} V_b$.

If every induced subgraph $\mathcal{G}(V_b)$ for $b \in J$ is connected and the quotient graph $\mathcal{Q}[\mathcal{G}]$ is connected, then $\mathcal{G}$ is connected.

#### Proposition 2

For graph $\mathcal{G} = (V,E)$, the following statements are equivalent:

- $$\mathcal{G}$$ is connected.
- For every partition of *V*, the quotient graph $\mathcal{Q}[\mathcal{G}]$ is connected.

## Properties of taboo-sets

We will repetadly use of the following terminology.

### Definition 1

- A finite set of strings $\mathbb {T}$ such that every $t \in \mathbb {T}$ satisfies $|t| \ge 2$ is called a **taboo-set**.
- Strings in $\mathbb {T}$ are called **taboos**.
- The **length of the longest taboo(s)** in $\mathbb {T}$ will be denoted by $M := \max \; \{ |t|\}_{t \in \mathbb {T}}$.
- A string is **taboo-free** if it does not contain any taboo of $\mathbb {T}$ as substring.
- $V_n(\mathbb {T})$ denotes the **set of taboo-free strings of length** *n* .
- $V_n^{s}(\mathbb {T})$ denotes the **set of strings in**  $V_n(\mathbb {T})$ **with suffix**  *s*.
- Similarly, $^sV_n(\mathbb {T})$ denotes all strings in $V_n(\mathbb {T})$ with prefix *s*.

With Definition 1 in mind, we can prove some simple properties of taboo-sets.

### Proposition 3

Given taboo-sets $\mathbb {T}_1$ and $\mathbb {T}_2$, it holds that:

- Set $\mathbb {T}_1 \bigcup \mathbb {T}_2$ is a taboo-set
- For $$n \in \mathbb {N}$$, $$V_n(\mathbb {T}_1) \bigcap V_n(\mathbb {T}_2) = V_n( \mathbb {T}_1 \bigcup \mathbb {T}_2).$$
- If for every $t_1 \in \mathbb {T}_1$ there exists $t_2 \in \mathbb {T}_2$ such that $t_2 \prec t_1$, then for any $n \in \mathbb {N}$, ${V_n(\mathbb {T}_2) \subseteq V_n(\mathbb {T}_1)}$.

### Proof

- Every $t \in \mathbb {T}_1 \bigcup \mathbb {T}_2$ has length at least 2, and thus $\mathbb {T}_1 \bigcup \mathbb {T}_2$ is a taboo-set.
- All strings $s \in V_n(\mathbb {T}_1) \bigcap V_n(\mathbb {T}_2)$ satisfy $t_1\not \prec s$ for all $t_1 \in \mathbb {T}_1$ and $t_2\not \prec s$ for all $t_2 \in \mathbb {T}_2$ this is equivalent to *s* satisfying $t \not \prec s$ for all $t \in \mathbb {T}_1 \bigcup \mathbb {T}_2$.
- Consider $s \in V_n(\mathbb {T}_2)$. Assume that $s \notin V_n(\mathbb {T}_1)$; then there exists $t_1 \in \mathbb {T}_1$ such that $t_1 \prec s$. But there also exists a $t_2 \in \mathbb {T}_2$ such that $t_2 \prec t_1$, and thus $t_2 \prec s$, a contradiction. Hence $s \in V_n(\mathbb {T}_1)$.$\square$

For a given *n* and $\mathbb {T}$, we can find a taboo-set $\mathbb {T}' \ne \mathbb {T}$ such that ${V_n(\mathbb {T}) = V_n(\mathbb {T}').}$ In this sense, taboo-sets are not unique, as we illustrate in the following proposition.

### Proposition 4

For a string *t* and $n \ge |t|+1$, it holds that

$$\begin{aligned} V_n( \{ t \} ) = V_n \Big ( (t \circ \varSigma ) \; \bigcup \; (\varSigma \circ t) \Big ). \end{aligned}$$

### Proof

- $\subseteq$ : Any taboo in $\mathbb {T}_1 := (t \circ \varSigma ) \; \bigcup \; (\varSigma \circ t)$ has $t \in \mathbb {T}_2 := \{ t \}$ as substring, and thus Proposition 3.c implies $V_n( \{ t \} ) \subseteq V_n ( (t \circ \varSigma ) \; \bigcup \; (\varSigma \circ t))$.
- $\supseteq$ : Assume that there exists an $s \in V_n ( (t\circ \varSigma ) \bigcup (\varSigma \circ t) )$ with $t \prec s$. Since $|s| = n$ and ${n \ge |t| + 1}$, the substring *t* is either preceded or followed by some symbol $a \in \varSigma .$ This contradicts ${\{ at, ta \} \subseteq (t \circ \varSigma ) \cup (\varSigma \circ t ).}$ $\square$

Proposition 4 implies that, for any $\mathbb {T}$, we can construct many taboo-sets $\mathbb {T}'$ such that $V_n(\mathbb {T}) = V_n(\mathbb {T}')$ as long as $n \ge \max (M, M')$, where *M* and $M'$ denote the length of the longest taboo in $\mathbb {T}$ and $\mathbb {T}',$ respectively.

### Example 2

If $\mathbb {T} = \mathbb {T}_1 \bigsqcup \mathbb {T}_2$ with $\mathbb {T}_2 = (t \circ \varSigma ) \cup (\varSigma \circ t )$, Proposition 3.a and 4 imply that $\mathbb {T}' := \mathbb {T}_1 \bigsqcup \{ t \}$ satisfies $V_{n}(\mathbb {T}) = V_n(\mathbb {T}')$ for any $n \ge M$. Repeating this process, we can construct a taboo-set $\mathbb {T}'$ such that $(t \circ \varSigma ) \bigcup \; (\varSigma \circ t ) \not \subseteq \mathbb {T}'$ for any string *t* and satisfying $V_{n}(\mathbb {T}) = V_n(\mathbb {T}')$ for any $n \ge M$.

Example 2 and Proposition 4 motivate the following definition.

### Definition 2

A taboo-set $\mathbb {T}$ is **minimal** if the following conditions hold:

- For every different $t_1, t_2 \in \mathbb {T}$, it holds that $t_1 \not \prec t_2$.
- For every $j \in [0, M-1]$ and $s \in V_j(\mathbb {T})$, set $(s \circ \varSigma ) \bigcup \; (\varSigma \circ s )$ is not a subset of $\mathbb {T}.$

Condition (a) is easy to justify: If string *AA* is a taboo, it is redundant that *AAA* be a taboo. Condition (b) avoids unnecessarily complicated taboo-sets. For example, using the four-nucleotide alphabet, taboo-set $\mathbb {T} = \{ AAA, AAC, AAG, AAT, CAA, GAA, TAA\}$ can be minimized as ${\mathbb {T}' = \{ AA \}}$. In general, one can minimize a taboo-set according to Example 2.

Since we want to study taboo-free strings of arbitrary lengths, we need conditions to concatenate taboo-free strings such that the concatenated sequence is taboo-free. The following result gives such a condition.

### Proposition 5

Given taboo-set $\mathbb {T}$, consider three strings $s_1, s_2,s_3$ such that $s_1s_2$ and $s_2s_3$ are taboo-free and $|s_2] \ge M-1$. Then $s := s_1s_2s_3$ is taboo-free.

### Proof

If $|s_1|=0$ and $|s_3| = 0$, then $s = s_2$ is taboo-free, as desired. Now assume either $|s_1|>0$ or $|s_3|>0$, yielding $n :=|s_1| + |s_2| + |s_3| \ge M$. For each $i \in [1, n-(M+1)]$, the fact that $|s_2| \ge M-1$ implies that either ${s[i, i + M-1]\prec s_1s_2}$ or ${s[i, i + M-1]\prec s_2s_3}$, hence each $s[i, i + M-1]$ is taboo-free and the result follows. $\square$

## Prefixes and suffixes of a taboo-free string

Given a taboo-free string *s*, the construction of set $V^s_{n}(\mathbb {T})$ for $n > |s|$ depends on which string *w* can be concatenated to the left side of *s*, such that $ws \in V_n(\mathbb {T})$. This motivates the following definition.

### Definition 3

Given a taboo-set $\mathbb {T}$, consider a taboo-free string *s* and $k \in \mathbb {N}_{0}$. The *k*-**prefixes** of *s* are the elements of the set $L^k(s)$, defined as

$$\begin{aligned} L^k(s) := \left\{ w \in \varSigma ^k \text { such that } ws \text { is taboo-free }\right\} = V^s_{|s|+k}(\mathbb {T})[1, k]. \end{aligned}$$

If $L^k(s) \ne \emptyset$, then we will say that *s* is *k*-**prefixable**.

Similarly, the *k*-**suffixes** of *s*, denoted $R^k(s)$, are the strings $w \in \varSigma ^k$ such that $sw \in V_{|s|+k}(\mathbb {T})$, that is, $R^k(s) := {^sV_{|s|+k}}(\mathbb {T})[|s|+1,|s|+k]$. When $R^k(s) \ne \emptyset$, we say that *s* is *k*-**suffixable**.

### Example 3

If $\varSigma = \{ A, C ,G, T\}$ and ${\mathbb {T} = \{ CAA, GAA, TAA\}}$, then ${L^1(AA) = \{ A\}}$ and ${L^2(AA) = \{ AA\}}$. Hence string *AA* is 1-prefixable and 2-prefixable. Moreover, ${R^1(AA) = \{ A,C,G,T\}}$, hence string *AA* is 1-suffixable.

By construction, given $s \in V_{|s|}(\mathbb {T})$, for any $k \in \mathbb {N}_{0}$ it holds that

1 $$\begin{aligned} V^s_{k + |s|}(\mathbb {T}) = L^k(s) \circ s. \end{aligned}$$

That is, $V_{k + |s|}^s(\mathbb {T})$ is $L^k(s)$ with *s* concatenated. Moreover, the following proposition shows that the *k*-prefixes of a string *s* induce a disjoint partition of the set $V^s_n(\mathbb {T})$.

### Proposition 6

Given a taboo-set $\mathbb {T}$ and a taboo-free string *s*, consider integers $k \in \mathbb {N}_{0}$ and ${n \ge k + |s|}$. It holds that

$$\begin{aligned} V_{n}^{s}(\mathbb {T}) = \bigsqcup _{w \in L^k(s)} V_n^{ws} (\mathbb {T}). \end{aligned}$$

That is, the set $V_{n}^{s}(\mathbb {T})$ can be partitioned into the disjoint sets of taboo-free strings of length *n* with suffix *ws*, where $w \in L^k(s)$.

### Proof

If *s* is not *k*-prefixable, then $L^k(s) = \emptyset$ and $V_{n}^{s}(\mathbb {T}) = \emptyset$, hence the equation holds. Otherwise, the inclusion $\supseteq$ is clear, while the $\subseteq$ follows from the fact that, for any string $w \in \varSigma ^k$ preceding the suffix *s*, this *w* must necessarily belong to $L^k(s)$. $\square$

Clearly, if a taboo-free string *s* is $k^*$-prefixable, then it is also *k*-prefixable for any integer $k < k^*$, while nothing can be said *a priori* about the case $k > k^*$. Consequently, we need to find conditions under which one can concatenate at least one symbol to the left of a taboo-free string. We will first introduce such taboo-sets in Definition 4 and then characterize prefixability in Proposition 7.

### Definition 4

A taboo-set $\mathbb {T}$ is called **left proper** if every $s \in V_M(\mathbb {T})$ is 1-prefixable. Analogously, $\mathbb {T}$ is **right proper** if every $s \in V_M(\mathbb {T})$ is 1-suffixable.

### Example 4

If $\varSigma = \{ A, C, G, T\}$ and $\mathbb {T} = \varSigma \circ A$, then $AC \in V_2(\mathbb {T})$ and *AC* is not 1-suffixable. Thus, $\mathbb {T}$ is not left proper.

### Proposition 7

Consider a left proper taboo-set $\mathbb {T}$ and a taboo-free string *s* such that one of the following conditions holds:

- $$|s| \ge M$$
- $|s| \le M-1$ and *s* is $(M-|s|)$-prefixable

Then *s* is *k*-prefixable for every $k \in \mathbb {N}$.

### Proof

If condition (a) applies, then the prefix $s[1, M] \in V_M(\mathbb {T})$ is 1-prefixable, because $\mathbb {T}$ is left proper. That is, there exists $a \in \varSigma$ with $as \in V_{1+|s|}(\mathbb {T})$. Proceeding analogously with (*as*)[1, *M*] , we infer that *s* is 2-prefixable. Continuing with this process, we deduce that *s* is *k*-prefixable for any $k \in \mathbb {N}$.

If condition (b) holds, then we can take any string in $V_{M}^s(\mathbb {T})$ and proceed as we did assuming (a). $\square$

We mainly study left proper taboo-sets due to Proposition 7, because the existence of arbitrary *k*-prefixes is necessary in many of our proofs. Analogous results for right proper taboo-sets are obtained by reversing the order of the symbols composing the string.

According to Proposition 7, if the length of a taboo-free string is at least *M*, then the taboo-free string can prefixed for arbitrary lengths. Otherwise, one needs to check the $(M-|s|)$-prefixability of this string. To that end, the following result comes in handy.

### Proposition 8

Consider a left proper taboo-set $\mathbb {T}$ and a taboo-free string *s*.

- If $|s| \le M-1$ and $s \notin {{\,\mathrm{suf}\,}}(V_M(\mathbb {T}))$, then $V^s_n(\mathbb {T}) = \emptyset$ for $n \ge M$.
- If either $|s| \ge M$ or $s \in {{\,\mathrm{suf}\,}}(V_M(\mathbb {T}))$, then $V^s_n(\mathbb {T}) \ne \emptyset$ for $n \ge \max (|s| , M)$.

### Proof

- If $0 \le |s| \le M-1$ and $s \notin {{\,\mathrm{suf}\,}}(V_M(\mathbb {T}))$, since ${{\,\mathrm{suf}\,}}(V_M^s(\mathbb {T})) \subseteq {{\,\mathrm{suf}\,}}(V_M(\mathbb {T}))$, it holds that ${V_M^s(\mathbb {T}) = \emptyset }$. This implies that $V^s_n(\mathbb {T}) = \emptyset$ for every $n \ge M$, because otherwise
  $$\begin{aligned}\emptyset \subsetneq V^s_n(\mathbb {T})[n-M+1,n] \subseteq V^s_M(\mathbb {T}),\end{aligned}$$
  which contradicts $${V_M^s(\mathbb {T}) = \emptyset }$$.
- If $|s| \ge M$, since $\mathbb {T}$ is left proper, Proposition 7.a implies that *s* is *k*-prefixable for every $k \in \mathbb {N}$. Thus, $V^s_n(\mathbb {T}) \ne \emptyset$. Similarly, if $s \in {{\,\mathrm{suf}\,}}(V_M(\mathbb {T}))$, then *s* is $(M-|s|)$-prefixable, and thus Proposition 7.b implies that *s* is *k*-prefixable for every $k \in \mathbb {N}$.$\square$

Note that, since the assumptions of Proposition 8.a are the negation of the assumptions of Proposition 8.b, in Proposition 8 we have proved that $V^s_n(\mathbb {T}) = \emptyset$ for $n \ge M$ **iff** string *s* satisfies $|s| \le M-1$ and $s \notin {{\,\mathrm{suf}\,}}(V_M(\mathbb {T}))$.

To study the connectivity of Hamming graphs $\varGamma ^s_n(\mathbb {T})$, we need to know whether two different strings have a *k*-prefix in common. Thus, we introduce the following.

### Definition 5

Given a taboo-set $\mathbb {T}$, we say that two taboo-free strings $s_1$ and $s_2$ (maybe of different length) are **left**  *k*-**synchronized** if ${L^k(s_1) \bigcap L^k(s_2) \ne \emptyset }$. If $R^k(s)\bigcap R^k(r) \ne \emptyset$, then we say that $s_1$ and $s_2$ are **right**  *k*-**synchronized**.

In words, two taboo-free strings are left *k*-synchronized if they are *k*-prefixable by at least one string *w*. Clearly, two taboo-free strings $s_1, s_2$ that are left $k^*$-synchronized are also left *k*-synchronized for any $k \le k^*$ (one simply has to “cut” the *k* symbols on the left of $L^{k^*}(s_1) \bigcap L^{k^*}(s_2)$). The following proposition states when we can also guarantee *k*-synchronization for $k > k^*$:

### Proposition 9

Consider a left proper taboo-set $\mathbb {T}$ and two taboo-free strings $s_1, s_2$, with length greater than zero, such that $s_1$ and $s_2$ are left $(M-1)$-synchronized. Then $s_1$ and $s_2$ are left *k*-synchronized for any $k \in \mathbb {N}$.

### Proof

If $k \le M -1$, then the assertion is true since $s_1$ and $s_2$ are $(M-1)$-synchronized.

For $k > M -1$, consider a string $w \in L^{M-1}(s_1) \bigcap L^{M-1}(s_2)$. We know that $ws_1$ and $ws_2$ are taboo-free strings with length at least *M*. Since $\mathbb {T}$ is left proper, Proposition 7.a applied to $ws_1$ and $ws_2$ implies that $ws_1$ and $ws_2$ are $k'$-prefixable for any $k' \in \mathbb {N}$. Therefore *w* is $k'$-prefixable for any $k' \in \mathbb {N}$. For any $k'$, take $x \in L^{k'}(w)$ and consider strings *xws* and *xwr*. The fact that $|w| = M-1$, together with the fact that *xw* and the pair $ws_1, ws_2$ are taboo-free, allows applying Proposition 5, hence $xws_1$ and $xws_2$ are also taboo-free.

It follows that $xw \in L^{M-1+k'}(s_1) \bigcap L^{M-1+k'}(s_2)$. With $k := M-1+k'$, the result follows for any $k > M-1$. $\square$

The following proposition provides a Hamming-distance based criterion to quickly decide whether two taboo-free strings of length *M* are left *k*-synchronized.

### Proposition 10

Consider a left proper taboo-set $\mathbb {T}$. If all pairs $s_1, s_2 \in V_M(\mathbb {T})$ with $d(s_1, s_2) = 1$ are left 1-synchronized, then all pairs $s_1, s_2 \in V_M(\mathbb {T})$ with $d(s_1, s_2) = 1$ are left *k*-synchronized for all $k \in \mathbb {N}_0$.

### Proof

Given any left 1-synchronized pair $s_1, s_2$ with $d(s_1, s_2) = 1$, there exists an $a \in \varSigma$ such that $as_1$ and $as_2$ are taboo-free. Since $(as_i)[1, M] \in V_M(\mathbb {T})$ for $i \in \{ 1, 2\}$ and the Hamming distance between these two strings is at most 1, $as_1, as_2$ are 1-synchronized, hence there exists a symbol $b \in \varSigma$ such that $bas_1$ and $bas_2$ are taboo-free, i.e. $s_1$ and $s_2$ are left 2-synchronized. Continuing with this process, it follows that $s_1$ and $s_2$ are *k*-synchronized. $\square$

We will now discuss conditions that allow increasing the string length of an entire set of taboo-free strings. To this end, consider two taboo-free strings $s_1, s$ and the set $V^{s_1s}_{n + |s_1| + |s|}(\mathbb {T})$. It is generally not true that $V^{s_1s}_{n + |s_1| + |s|}(\mathbb {T}) = V^{s_1}_{n+|s_1|}(\mathbb {T})\circ s$, because the concatenation of *s* to a taboo-free string from $V^{s_1}_{n+|s_1|}(\mathbb {T})$ can create a taboo string around the junction of both strings. For the remainder of this section we will discuss when the equality holds.

### Definition 6

For a taboo-set $\mathbb {T}$ and a taboo-free string *s*, we define the **length of the longest taboo suffix-prefix match** as

$$\begin{aligned} k_s := \max \Big \{ i \in [0, |s|] \; \Big | \; s[1,i]\in {{\,\mathrm{suf}\,}}(\mathbb {T}) \Big \}, \end{aligned}$$

i.e. $k_s$ denotes the length of the longest prefix of *s* being a proper suffix of a taboo.

Note that the length $k_s$ is well defined, because $s[1,0]=e \in {{\,\mathrm{suf}\,}}(\mathbb {T})$, hence ${k_s \in [0, \min \; (M-1, |s|)].}$ Using this length $k_s$, in Proposition 11 we give conditions implying that equality $V^{s_1s}_{n + |s_1| + |s|}(\mathbb {T}) = V^{s_1}_{n+|s_1|}(\mathbb {T})\circ s$ holds.

### Proposition 11

For a taboo-set $\mathbb {T}$ and a taboo-free string *s*, the following holds:

- Take $w \in \varSigma ^{M-1}$ such that $ws \in V_{M-1+|s|} (\mathbb {T})$. Then for any $n \ge M-1$,
  $$\begin{aligned}V_{n+|s|}^{ws}(\mathbb {T}) = V_{n}^{w}(\mathbb {T}) \circ s. \end{aligned}$$
- For any $n \in \mathbb {N}_0$ it holds that
  $$\begin{aligned} V_{n+|s|}^s(\mathbb {T}) = V_{n+k_s}^{s[1, k_s]}(\mathbb {T}) \circ s[k_s+1, |s|]. \end{aligned}$$

### Proof

- The inclusion $\subseteq$ is clear. The inclusion $\supseteq$ follows from the fact that, if we are given $rw \in V^w_{n} (\mathbb {T})$ such that $ws \in V_{M-1+j} (\mathbb {T})$, since $|w| = M-1$, Proposition 5 yields that the concatenated string *rws* is taboo-free.
- The result is obvious if $|s| = 0$ or $n = 0$, hence assume $|s| > 0$ and $n > 0$.
  Clearly $V_{n+|s|}^s(\mathbb {T}) \subseteq V_{n+k_s}^{s[1, k_s]}(\mathbb {T}) \circ \; s[k_s+1, |s|]$. For $r \in V_{n}(\mathbb {T})$, consider $r s[1, k_s] \in V_{n+k_s}^{s[1, k_s]}(\mathbb {T})$. We need to prove that the string
  $$\begin{aligned}r s[1, k_s]s[k_s+1, |s|] = rs \end{aligned}$$
  is taboo-free. But otherwise, since $r s[1, k_s]$ and *s* are taboo-free, there would exist integers *c*, *d* such that $1 \le c \le |r| \le |r| + k_s < d \le |r| + |s|$ and $(rs)[c, d] \in \mathbb {T}$. Take $k^* := d - |r| > k_s$, which yields $s[1, k^*] \in {{\,\mathrm{suf}\,}}(\mathbb {T})$, contradicting the maximality of $k_s$. Hence *rs* is taboo-free, as desired. Note that the same argument applies if $k_s=0$. $\square$

From Proposition 11.b we obtain two corollaries.

### Corollary 12

Given a taboo-set $\mathbb {T}$ and a taboo-free string *s*, for any $k \in \mathbb {N}_0$ it holds that

$$\begin{aligned}L^k(s) = L^k(s[1, k_s]).\end{aligned}$$

### Proof

By construction, $L^k(s) = V^s_{|s|+k}(\mathbb {T})[1, k].$ Proposition 11.b yields

$$\begin{aligned} V^s_{k+|s|}(\mathbb {T})[1, k]&= \Big ( V_{k+k_s}^{s[1, k_s]}(\mathbb {T}) \circ s[k_s+1, |s|] \Big ) [1, k] \\&= V_{k+k_s}^{s[1, k_s]}(\mathbb {T}) [1, k]= L^k(s[1,k_s]). \end{aligned}$$

$$\square $$

### Corollary 13

For a taboo-set $\mathbb {T}$ and for any pair of taboo-free strings $s_1$ and $s_2$, the following statements are equivalent for all $k \in \mathbb {N}_0$:

- $s_1$ and $s_2$ are left *k*-synchronized
- $s_1 [1,k_{s_1}]$ and $s_2[1,k_{s_2}]$ are left *k*-synchronized.

### Proof

Strings $s_1$ and $s_2$ are left *k*-synchronized iff ${L^k(s_1) \bigcap L^k(s_2) \ne \emptyset .}$ We just have to apply Corollary 12. $\square$

Thus, the string $s[1,k_s]$, which is the longest prefix of *s* that matches a proper suffix of the taboos, provides all the information we need to construct $V^s_n(\mathbb {T})$ or $L^k(s)$.

## Isomorphisms between taboo-free Hamming graphs

Here we will discuss isomorphism between Hamming graphs. Let us first introduce the formal definition of a taboo-free Hamming graph.

### Definition 7

*The taboo-free Hamming graph of length* *n*, ${\varGamma _n(\mathbb {T}) := (V_n(\mathbb {T}), E_n(\mathbb {T}))}$, is the graph with vertex set $V_n(\mathbb {T})$ such that two vertices $u, v \in V_n(\mathbb {T})$ are adjacent if their Hamming distance equals 1, that is, $e = \{ u,v\} \in E_n(\mathbb {T})$ iff $d(u, v) = 1.$ Analogously, $\varGamma ^s_n(\mathbb {T})$ is the Hamming graph with vertex set $V^s_n(\mathbb {T})$.

Examples of disconnected Hamming graphs are given in Figs. 1 and 2. When dealing with taboo-free Hamming graphs, the following proposition is a simple way to establish graph isomorphisms.

### Proposition 14

Consider a taboo-set $\mathbb {T}$, a taboo-free string *s* and a taboo-free string *w* satisfying $ws \in V_{|w|+|s|}(\mathbb {T})$. If $V^{ws}_{n+|s|}(\mathbb {T}) = V^{w}_{n}(\mathbb {T}) \circ s$ for some $n \ge |w|$, then $\varGamma ^{ws}_{n+|s|}(\mathbb {T})$ and $\varGamma ^w_{n} (\mathbb {T})$ are isomorphic.

### Proof

By assumption, the vertex set of $\varGamma ^{ws}_{n+|s|}(\mathbb {T})$ is $V^{ws}_{n+|s|}(\mathbb {T}) = V^{w}_{n}(\mathbb {T}) \circ s$. Thus, the map

$$\begin{aligned} f :V_{n}^{w}(\mathbb {T}) \circ s&\rightarrow V^{w}_{n}(\mathbb {T})\\ rs&\mapsto r \end{aligned}$$

is well defined and bijective. Moreover, *f* is an edge-preserving bijection: Given any pair of strings $r_1, r_2 \in \varSigma ^n$ and any string $s \in \varSigma ^{|s|}$, then $d(r_1, r_2) = 1$ iff $d(r_1s, r_2s) = 1$. $\square$

Propositions 14 and 11.a imply that, for a taboo-free string *s* with $|s| \ge M$, the graphs $\varGamma ^s_{n+|s|}(\mathbb {T})$ and $\varGamma ^{s[1, M-1]}_{n+M-1}(\mathbb {T})$ are isomorphic. Furthermore Proposition 11.b implies that ${\varGamma ^s_{n+|s|}(\mathbb {T}) \simeq \varGamma ^{s[1, k_s]}_{n + k_s}(\mathbb {T}),}$ which can be stated as follows.

### Proposition 15

Consider a taboo-set $\mathbb {T}$ and a taboo-free string *s*. There exists a unique ${w \in {{\,\mathrm{suf}\,}}(\mathbb {T})}$ such that $w = s[1, k_s]$. Moreover, for any $n \ge 0$,

$$\begin{aligned} \varGamma _{ n + |s|}^s(\mathbb {T}) \simeq \varGamma _{n + |w|}^w(\mathbb {T}).\end{aligned}$$

Proposition 15 does not describe in which cases $V_{n+|s|}^s(\mathbb {T}) = \emptyset$. However, if $\mathbb {T}$ is left proper, Proposition 8 implies that this happens iff $|s| \le M-1$ and $s \notin {{\,\mathrm{suf}\,}}(V_M(\mathbb {T}))$. This suggests that we can state a version of Proposition 15 for left proper $\mathbb {T}$. But first, due to our interest in taboo-free strings of length *M*, we introduce the following.

### Definition 8

Given a left proper taboo-set $\mathbb {T}$, **the long suffix classification** ${{\,\mathrm{lsc}\,}}(\mathbb {T})$ is defined as

$$\begin{aligned} {{\,\mathrm{lsc}\,}}(\mathbb {T}) := \{ w \in {{\,\mathrm{suf}\,}}(\mathbb {T}) \text { such that } \exists s\in V_M(\mathbb {T}) \text { satisfying } s[1, k_s] = w\}, \end{aligned}$$

that is, ${{\,\mathrm{lsc}\,}}(\mathbb {T})$ is the set of all suffixes of taboos that are the longest prefix of at least one taboo-free string of length *M*.

### Example 5

If $\varSigma _1 = \{ A, C, G, T\}$ and $\mathbb {T}_1 = \{ AA,\; CC, \; GG, \;TT \}$, then

$$\begin{aligned} {{\,\mathrm{lsc}\,}}(\mathbb {T}_1) \subseteq {{\,\mathrm{suf}\,}}(\mathbb {T}_1) = \{ A, \; C,\; G,\; T, \; e\} = \varSigma _1 \; \bigcup \; \{ e \} .\end{aligned}$$

For any $s \in V_2(\mathbb {T}_1)$, we see $k_s > 0$, hence $e \notin {{\,\mathrm{lsc}\,}}(\mathbb {T}_1)$. Moreover,

$$\begin{aligned} \{ AC, CG, GT, TA \} \subseteq V_2(\mathbb {T}_1),\end{aligned}$$

yielding ${{\,\mathrm{lsc}\,}}(\mathbb {T}_1) = \varSigma _1$. If we consider $\varSigma _2 := \{ A, C, G, T, C'\}$, where $C'$ could represent a 5-methylcytosine, and $\mathbb {T}_2 := \mathbb {T}_1$, then string $s = C'A$ satisfies $k_s = 0$, hence ${{\,\mathrm{lsc}\,}}(\mathbb {T}_2) = {{\,\mathrm{suf}\,}}(\mathbb {T}_2)$.

The following theorem classifies graphs $\varGamma ^s_n(\mathbb {T})$ for left proper $\mathbb {T}$.

### Theorem 16

Consider a left proper taboo-set $\mathbb {T}$ and a taboo-free string *s* such that either $|s| \ge M$ or ${s \in {{\,\mathrm{suf}\,}}(V_M(\mathbb {T}))}$. Then a unique ${w \in {{\,\mathrm{suf}\,}}(V_M(\mathbb {T})) \bigcap {{\,\mathrm{suf}\,}}(\mathbb {T})}$ exists such that ${w = s[1, k_s]}$, which satisfies ${\varGamma _{ n + |s|}^s(\mathbb {T}) \simeq \varGamma _{n + |w|}^w(\mathbb {T})}$ for ${n \ge 0}$. Moreover, if $|s| \ge M$, then ${w \in {{\,\mathrm{lsc}\,}}(\mathbb {T})}$.

### Proof

Proposition 8.b yields $V^s_{n+|s|}(\mathbb {T}) \ne \emptyset$ for $n \ge 0$, while $\varGamma ^s_{n+|s|}(\mathbb {T}) \simeq \varGamma ^{s[1, k_s]}_{n + k_s}(\mathbb {T})$ for $n \ge 0$ follows from Proposition 15. Hence we can set $w:= s[1, k_s]$, which by definition belongs to ${{\,\mathrm{suf}\,}}(\mathbb {T})$. Since by assumption either $|s| \ge M$ or ${s \in {{\,\mathrm{suf}\,}}(V_M(\mathbb {T}))}$, it follows from Proposition 7 that *s* is *k*-prefixable for any *k*, and thus also $w:= s[1, k_s]$ is *k*-prefixable. We consider $x \in L^{M-k_s}(w)$, which satisfies ${xw \in V_M(\mathbb {T})}$. Therefore $w = (xw)[M-k_s+1, M] \in {{\,\mathrm{suf}\,}}(V_M(\mathbb {T}))$. All in all, ${w \in {{\,\mathrm{suf}\,}}(V_M(\mathbb {T})) \bigcap {{\,\mathrm{suf}\,}}(\mathbb {T})}$. This *w* is trivially unique since $k_s$ is uniquely determined given *s*.

As for the case $|s| \ge M$, the fact that ${s[1, M] \in V_M(\mathbb {T})}$ and the definition of ${{\,\mathrm{lsc}\,}}(\mathbb {T})$ implies that $w \in {{\,\mathrm{lsc}\,}}(\mathbb {T})$. $\square$

In formal terms, Theorem 16 states that the equivalence relation “being isomorphic” divides all graphs $\varGamma ^s_{n+|s|}(\mathbb {T})$ into equivalence classes. The representative of each class is a graph $\varGamma ^w_{n+|w|}(\mathbb {T})$, where $w \in {{\,\mathrm{suf}\,}}(V_M(\mathbb {T})) \bigcap {{\,\mathrm{suf}\,}}(\mathbb {T})$. When $|s| \ge M$, string *w* belongs to ${{\,\mathrm{lsc}\,}}(\mathbb {T})$. This is why ${{\,\mathrm{lsc}\,}}(\mathbb {T})$ is called the long suffix classification.

To efficiently compute ${{\,\mathrm{lsc}\,}}(\mathbb {T})$, we recommend that $\mathbb {T}$ be minimal. Theorem 16 implies that

2 $$\begin{aligned} {{\,\mathrm{lsc}\,}}(\mathbb {T}) \subseteq {{\,\mathrm{suf}\,}}(V_M(\mathbb {T})) \bigcap {{\,\mathrm{suf}\,}}(\mathbb {T}), \end{aligned}$$

and thus we define the **short suffix classification** as

3 $$\begin{aligned} {{\,\mathrm{ssc}\,}}(\mathbb {T}) := \Big ( {{\,\mathrm{suf}\,}}(V_M(\mathbb {T})) \bigcap {{\,\mathrm{suf}\,}}(\mathbb {T}) \Big ) - {{\,\mathrm{lsc}\,}}(\mathbb {T}). \end{aligned}$$

The set ${{\,\mathrm{ssc}\,}}(\mathbb {T})$ is called short suffix classification because only when $|s| < M$ it can happen that a graph $\varGamma ^s_{n+|s|}(\mathbb {T})$ is represented by a graph $\varGamma ^w_{n+|w|}(\mathbb {T})$ with $w \in {{\,\mathrm{ssc}\,}}(\mathbb {T})$. Note that, if a string *w* satisfies the condition ${|w| < M-1}$ and ${w \circ R^i(w) \subseteq {{\,\mathrm{suf}\,}}(\mathbb {T})}$ for some ${i \in [1, M-1-|w|]}$, then any $s \in w \circ R^i(w)$ satisfies ${s[1, k_s+i] \in {{\,\mathrm{suf}\,}}(\mathbb {T})}$, hence ${w \notin {{\,\mathrm{lsc}\,}}(\mathbb {T})}$. This property is used in the following example.

### Example 6

If $\varSigma _1 = \{ A, C, G, T\}$ and ${\mathbb {T}_1 = \{ AA,\; CC, \; GG, \;TT \}}$, then it is clear that ${e \in {{\,\mathrm{suf}\,}}(V_M(\mathbb {T})) \bigcap {{\,\mathrm{suf}\,}}(\mathbb {T})}$, because the empty string *e* belongs to both sets. Moreover, ${e \not \in {{\,\mathrm{lsc}\,}}(\mathbb {T}_1)}$ due to ${e \circ \varSigma \subseteq {{\,\mathrm{suf}\,}}(\mathbb {T}_1)}$. Therefore $e \in {{\,\mathrm{ssc}\,}}(\mathbb {T}).$

## Connectivity of taboo-free Hamming graphs

We will make extensive use of the quotient graph to study the connectivity of taboo-free Hamming graphs. Before we start with the technicalities, we briefly describe our initial strategy.

For a Hamming graph $\varGamma _{n+j} (\mathbb {T})$, let us consider two different subsets of its vertex set, namely $V^{s_b}_{n+j}(\mathbb {T})$ and $V^{s_c}_{n+j}(\mathbb {T})$, where $s_b, s_c \in V_j(\mathbb {T})$. These two subsets are disjoint, so we can use the quotient graph $\mathcal {Q}[\varGamma _{n+j} (\mathbb {T})]$ to make each of them collapse in a single vertex, represented respectively by $s_b$ and $s_c$. We will prove in Proposition 17 that $s_b$ and $s_c$ are adjacent in $\mathcal {Q}[\varGamma _{n+j}(\mathbb {T})]$ iff strings $s_b$ and $s_c$ have Hamming distance 1 and are left *n*-synchronized. This is specially interesting, because we know from Proposition 9 that two left $(M-1)$-synchronized strings are left *n*-synchronized for any $n \in \mathbb {N}$. Thus, it is enough to know that $s_b, s_c$ are adjacent in $\mathcal {Q}[\varGamma _{(M-1)+j}(\mathbb {T})]$ to claim that $s_b, s_c$ are adjacent in all partition graphs $\mathcal {Q}[\varGamma _{n+j}(\mathbb {T})]$ for $n \in \mathbb {N}$ (that is the essential content of Lemma 18). More formally, we have the following results.

### Proposition 17

Given taboo-set $\mathbb {T}$, $j \in \mathbb {N}_0$ and $n \in \mathbb {N}_0$, consider graph $\varGamma _{n+j}(\mathbb {T})$ and a subset $S \subseteq V_{n+j}(\mathbb {T})$ partitioned as $S = \bigsqcup _{b \in J} V^{s_b}_{n+j}(\mathbb {T})$, where $s_b$ are taboo-free strings of length *j*. Consider moreover the quotient graph ${\mathcal{Q}[\varGamma _{n+j}(\mathbb {T})(S)] = \{ J, E_J\}}$, where $\varGamma _{n+j}(\mathbb {T})(S)$ denotes the graph induced by *S* in $\varGamma _{n+j}(\mathbb {T})$.

In these conditions, a pair of vertices $b, c \in J$ is connected by an edge $\{ b, c \} \in E_J$ iff the pair $s_b$, $s_c$ is left *n*-synchronized and $d(s_b, s_c) = 1$.

### Proof

By definition, *b* and *c* are adjacent in $\mathcal{Q}[\varGamma _{n+j}(\mathbb {T})(S)]$ iff in graph $\varGamma _{n+j}(\mathbb {T})$ an edge connects a vertex in $V^{s_b}_{n+j}(\mathbb {T})$ with a vertex in $V^{s_c}_{n+j}(\mathbb {T})$. Since ${d(s_b, s_c) \ge 1}$, this edge exists iff $d(s_b, s_c) = 1$ and there exists $s \in V_n(\mathbb {T})$ such that ${ss_b, ss_c \in V_{n+j}(\mathbb {T})}$. The last condition is the definition of $s_b$ and $s_c$ being left *n*-synchronized. $\square$

The combination of Propositions 17 and 9 gives the following lemma.

### Lemma 18

Given a left proper taboo-set $\mathbb {T}$, a taboo-free string *s* and $k \in \mathbb {N}$, consider, for any $n \ge |s|+k$, partition ${V^s_{n}(\mathbb {T}) = \bigsqcup _{w \in L^{k}(s)} V^{ws}_{n}(\mathbb {T})}$ and quotient graph $\mathcal{Q}[\varGamma ^s_n(\mathbb {T})] = (L^k(s), E_{L^k(s)})$. Then it holds that

$$\begin{aligned} \mathcal{Q}\left[ \varGamma ^s_{|s|+k}(\mathbb {T})\right]&\supseteq \mathcal{Q}\left[ \varGamma ^s_{|s|+k+1}(\mathbb {T})\right] \supseteq \cdots \supseteq \mathcal{Q}\left[ \varGamma ^s_{|s| + k + M-1}(\mathbb {T})\right] \\&= \mathcal{Q}\left[ \varGamma ^s_{|s| + k + M}(\mathbb {T})\right] = \mathcal{Q}\left[ \varGamma ^s_{|s| + k + M+1}(\mathbb {T})\right] = \dots . \end{aligned}$$

If $\mathcal{Q}[\varGamma ^s_{|s| + k + M-1}(\mathbb {T})]$ is connected, then $\mathcal{Q}[\varGamma ^s_{n}(\mathbb {T})]$ is connected for $n \ge |s|+k$.

### Proof

For some $n_0 \ge |s| + k$, consider an edge $\{w_b, w_c\}$ of graph $\mathcal{Q}[\varGamma ^s_{n_0}(\mathbb {T})]$, where $w_b, w_c \in L^k(s)$. We set $s_b := w_bs$ and $s_c := w_cs$. Proposition 17 implies that $w_b$ and $w_c$ are adjacent in $\mathcal{Q}[\varGamma _{n_0}^s(\mathbb {T})]$ iff $s_b$ and $s_c$ are are left $(n_0-|s|-k)$-synchronized and $d(w_b, w_c) = 1$. Since $s_b$ and $s_c$ are left $(n_0-|s|-k)$-synchronized, they are also left $(n-|s|-k)$-synchronized for any $n \le n_0$, and thus $w_b$ and $w_c$ are adjacent in $\mathcal{Q}[\varGamma _{n}^s(\mathbb {T})]$ for $|s| + k \le n \le n_0$. Hence the decreasing chain of quotient graphs is proven.

Now we will prove that this chain stabilizes after ${n = |s| + k + M-1}$. If ${n_0-|s|-k = M-1}$, then, according to Proposition 9, $w_b$ and $w_c$ are left *k*-synchronized for arbitrary *k*, and thus Proposition 17 implies that $w_b$ and $w_c$ are adjacent in $\mathcal {Q}[\varGamma _n^s(\mathbb {T})]$ for arbitrary $n \ge |s| + k$. All in all, $\mathcal {Q}[\varGamma _{n_0}^s(\mathbb {T})]$ and $\mathcal {Q}[\varGamma _n^s(\mathbb {T})]$ have the same edges, as desired.

Regarding connectivity, given graphs $G_1$ and $G_2$ with the same vertex set $V_1 = V_2$ such that $G_1 \subseteq G_2$, if subgraph $G_1$ is connected, then $G_2$ is connected. $\square$

**Fig. 4:**
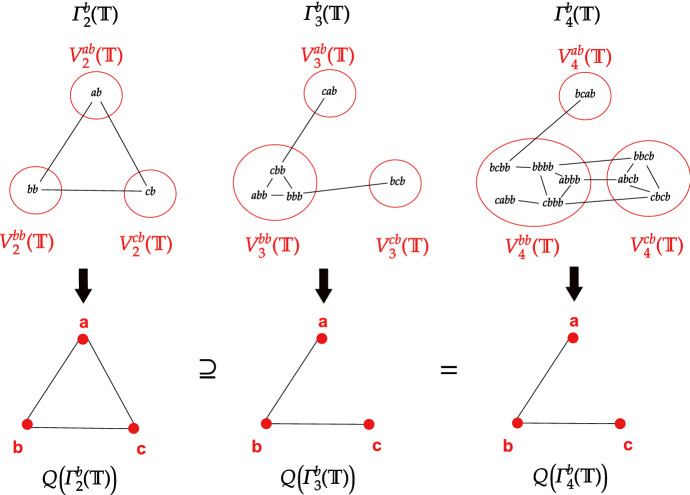
Visualization of Lemma 18 for $\varSigma = \{ a,b,c\}$, $\mathbb {T} = \{ ba, aa, ac, cc\}$, $s = b$ and $k = 1$. It holds that $L^1(b) = \{ a, b, c\}$

Figure 4 visualizes Lemma 18 for alphabet $\varSigma = \{ a,b,c\}$, taboo-set $\mathbb {T} = \{ ba, aa, ac, cc\}$ (which is left proper), suffix $s = b$ and $k = 1$.

We are finally ready to study the connectivity of graphs $\varGamma _n^s(\mathbb {T})$ for $|s| \ge M$. Let us begin with the following lemma.

### Lemma 19

Given a left proper $\mathbb {T}$, for any $w \in V_M(\mathbb {T})$ consider the set $V^w_{2M}(\mathbb {T})$ and partition

$$\begin{aligned}V^w_{2M}(\mathbb {T}) = \bigsqcup _{a \in L^1(w)} V^{aw}_{2M}(\mathbb {T}),\end{aligned}$$

inducing the quotient graph $\mathcal{Q}[ \varGamma ^w_{2M}(\mathbb {T})] = (L^1(w), E_{L^1(w)}).$ Then the following statements are equivalent:

- For every $w \in V_M(\mathbb {T})$, $\mathcal{Q}[ \varGamma ^w_{2M}(\mathbb {T})]$ is connected.
- For every $w \in V_M(\mathbb {T})$ and integer $n \ge M$, $\varGamma ^w_n(\mathbb {T})$ is connected.

### Proof

Proposition 2 states that, in a connected graph, every quotient graph is connected, and thus (b) implies (a) by considering $n = 2M$.

Now we prove by induction that (a) implies (b). For $n = M$ and $w \in V_M(\mathbb {T})$, we have that

$$\begin{aligned} V^w_{M}(\mathbb {T}) = \{ w \}, \end{aligned}$$

hence $\varGamma ^w_{M}(\mathbb {T})$ is connected. For the inductive step, assume that $\varGamma ^w_n(\mathbb {T})$ is connected for every $w \in V_M(\mathbb {T})$ and up to an integer $n \ge M$. We will prove that also every $\varGamma ^w_{n+1}(\mathbb {T})$ is connected. Consider

$$\begin{aligned} V^w_{n+1}(\mathbb {T}) = \bigsqcup _{a \in L^1(w)} V^{aw}_{n+1}(\mathbb {T}). \end{aligned}$$

Let us write *w* separating the first $M-1$ symbols from the last one, that is $w= r c$ for ${r \in \varSigma ^{M-1}}$ and $c \in \varSigma .$ Then for any $a \in L^1(w)$, $V^{aw}_{n+1}(\mathbb {T}) = V^{arc}_{n+1}(\mathbb {T})$. Since $|r| = M-1$, Proposition 11.a implies $V^{arc}_{n+1}(\mathbb {T}) = V^{ar}_{n}(\mathbb {T}) \circ c$, while the isomorphism established in Proposition 14 yields

$$\begin{aligned} \varGamma ^{aw}_{n+1}(\mathbb {T}) = \varGamma ^{arc}_{n+1}(\mathbb {T}) \simeq \varGamma ^{ar}_{n}(\mathbb {T}).\end{aligned}$$

Thus, every $\varGamma ^{aw}_{n+1}(\mathbb {T})$ is connected, because the induction hypothesis implies that $\varGamma ^{ar}_{n}(\mathbb {T})$ is connected since $ar \in V_M(\mathbb {T})$. To prove that graph $\varGamma _{n+1}^w(\mathbb {T})$ is connected, it remains to apply Proposition 1, so we need to prove that the quotient graph induced by partition $V^w_{n+1}(\mathbb {T}) = \bigsqcup _{a \in L^1(w)} V^{aw}_{n+1}(\mathbb {T})$, namely $\mathcal {Q}[\varGamma ^w_{n+1} (\mathbb {T})]$, is connected.

We know that, given partition $V^w_{2M}(\mathbb {T}) = \bigsqcup _{a \in L^1(w)} V^{aw}_{2M}(\mathbb {T})$, the quotient graph $\mathcal {Q}[ \varGamma ^w_{2M}(\mathbb {T})]$ is connected. Applying Lemma 18 with $s = w$ and $k = 1$, we get the following chain of inclusions:

$$\begin{aligned} \mathcal{Q}\left[ \varGamma ^w_{M+1}(\mathbb {T})\right]&\supseteq \mathcal{Q}\left[ \varGamma ^w_{M+2}(\mathbb {T})\right] \supseteq \cdots \supseteq \mathcal{Q}\left[ \varGamma ^w_{2M}(\mathbb {T})\right] \\&= \mathcal{Q}\left[ \varGamma ^w_{2M+1}(\mathbb {T})\right] = \mathcal{Q}\left[ \varGamma ^w_{2M+2}(\mathbb {T})\right] = \dots . \end{aligned}$$

Since $\mathcal{Q}[ \varGamma ^w_{2M}(\mathbb {T})]$ is connected, every quotient graph of the chain of inclusions is connected, as shown in Lemma 18. In particular, graph $\mathcal{Q}[\varGamma ^w_{n+1}(\mathbb {T})]$ is an element of the chain of inclusions because $n +1 \ge M+1$, so it is connected, as desired. $\square$

Lemma 19 is very interesting: We wanted to characterize the connectivity of graphs $\varGamma _n^s(\mathbb {T})$ for $s \in V_M(\mathbb {T})$ and $n \ge M$. We have proved that it is enough to study a finite number of graphs, namely $\mathcal{Q}[\varGamma ^w_{2M}(\mathbb {T})]$ for $s \in V_M(\mathbb {T})$, that is, $|V_M(\mathbb {T})|$ graphs. Let us summarize the connectivity results that follow from Lemma 19 and Theorem 16.

### Proposition 20

Given a left proper $\mathbb {T}$, the following statements are equivalent:

- For any taboo-free string *s* with $|s| \ge M$ and any integer $n \ge |s|$, $\varGamma ^s_n(\mathbb {T})$ is connected.
- For any $w \in V_M(\mathbb {T})$ and any integer $n \ge M$, $\varGamma ^w_n(\mathbb {T})$ is connected.
- For any $r \in {{\,\mathrm{lsc}\,}}(\mathbb {T})$, $\varGamma ^r_{M + |r|}(\mathbb {T})$ is connected.
- For any $r \in {{\,\mathrm{lsc}\,}}(\mathbb {T})$, the partition $V^r_{M + |r|}(\mathbb {T}) = \bigsqcup _{a \in L^1(r)} V^{ar}_{M + |r|}(\mathbb {T})$ induces a connected partition graph $\mathcal{Q}[ \varGamma ^r_{M + |r|}(\mathbb {T})].$

### Proof

Implication (a) $\Rightarrow$ (b) is obvious, while (b) $\Rightarrow$ (a) is proven as follows: Given $V_{n}^s(\mathbb {T})$, where *s* is a taboo-free string with $|s| \ge M$, Proposition 11.a implies that ${V_{n}^s(\mathbb {T}) = V_{n}^{s[1, M-1]}(\mathbb {T}) \circ s[M, j].}$ Since ${s[M, j] = s[M, M] s[M+1, j]}$, applying Proposition 11.a again we have ${V_{n}^s(\mathbb {T}) = V_{n}^{s[1, M]}(\mathbb {T}) \circ s[M+1, j]}$. Proposition 14 yields the isomorphism $\varGamma ^s_{n+j}(\mathbb {T}) \simeq \varGamma ^{s[1, M]}_{n+M}(\mathbb {T})$, and $\varGamma ^{s[1, M]}_{n+M}(\mathbb {T})$ is connected due to ${s[1, M] \in V_M(\mathbb {T})}$ and the assumption of (b). Thus, statements (a) and (b) are equivalent.

Implication (b) $\Rightarrow$ (c) is consequence of Theorem 16. Moreover, (c) $\Rightarrow$ (d) follows from Proposition 2. It remains to prove (d) $\Rightarrow$ (b), which we do as follows. Corollary 12 implies $L^1(w) = L^1(w[1, k_w])$. Moreover, for any $w \in V_M(\mathbb {T})$ and ${a,b \in L^1(w)}$, we claim that the following statements are equivalent:

(i) Strings *aw* and *bw* are left *k*-synchronized.
(ii) Strings $aw[1, k_w]$ and $bw[1, k_w]$ are left *k*-synchronized.

Indeed, the implication (i) $\Rightarrow$ (ii) is obvious, so let us prove (ii) $\Leftarrow$ (i). Given a taboo-free string $s \in V_j(\mathbb {T})$ such that $saw[1, k_w]$ and $sbw[1, k_w]$ are taboo-free, we want to prove that also *saw* and *sbw* are taboo-free. But if that were not the case, it would be the consequence of either $(saw)[c,d]\in \mathbb {T}$ or $(sbw)[c,d] \in \mathbb {T}$ for some integers $1 \le c \le j < j+1+k_w \le d \le j + 1 + M$. However, that contradicts the maximality of $k_w$, yielding $\text {ii}) \Leftarrow \text {i}$.

Our previous claim and Proposition 17 imply that, if $r = w[1, k_w]$ for some ${w \in V_M(\mathbb {T})}$, given partition $V^{r}_{M + |r|}(\mathbb {T}) = \bigsqcup _{a \in L^1(r)} V^{ar}_{M + |r|}(\mathbb {T})$, it holds that

$$\begin{aligned} \mathcal{Q}\left[ \varGamma ^r_n(\mathbb {T})\right] \simeq \mathcal{Q}\left[ \varGamma ^w_n(\mathbb {T})\right] . \end{aligned}$$

Theorem 16 implies that, for every $w \in V_M(\mathbb {T})$, there exists ${r = w[1, k_w] \in {{\,\mathrm{lsc}\,}}(\mathbb {T})}$. Applying Lemma 19, finally (d) $\Rightarrow$ (b) follows. $\square$

It is worth noticing how simpler the connectivity problem has become. Initially, we were studying whether every $\varGamma _n^s(\mathbb {T})$ with $|s| \ge M$ is connected, obtaining in Lemma 19 that this is equivalent to the connectivity of graphs $\varGamma _{2M}^w(\mathbb {T})$ for $w \in V_M(\mathbb {T})$, which are $|V_M(\mathbb {T})|$ graphs. Now we see, using Proposition 20 and the fact that ${{\,\mathrm{lsc}\,}}(\mathbb {T}) \subseteq {{\,\mathrm{suf}\,}}(\mathbb {T})$, that we only need to prove the connectivity of ${|{{\,\mathrm{lsc}\,}}(\mathbb {T})| \le |{{\,\mathrm{suf}\,}}(\mathbb {T})| \le (M-1)|\mathbb {T}| + 1}$ graphs, namely either $\mathcal{Q}[ \varGamma ^r_{M + |r|}(\mathbb {T})]$ or $\varGamma ^r_{M + |r|}(\mathbb {T})$ for $r \in {{\,\mathrm{lsc}\,}}(\mathbb {T})$. We give an example.

### Example 7

Take $\varSigma = \{ A, C, G, T\}$ and $\mathbb {T} = \{ AA,\; CCC\}$, which is left proper. Using Proposition 20, since $M = 3$ and ${{\,\mathrm{lsc}\,}}(\mathbb {T}) = {{\,\mathrm{suf}\,}}(\mathbb {T}) = \{ e, A, \; C,\; CC\},$ the connectivity of graphs

$$\begin{aligned} \varGamma ^e_{3}(\mathbb {T}), \; \varGamma ^{A}_{4}(\mathbb {T}), \; \varGamma ^{C}_{4}(\mathbb {T}), \; \varGamma ^{CC}_{5}(\mathbb {T})\end{aligned}$$

implies that any $\varGamma ^w_n(\mathbb {T})$ with $w \in {{\,\mathrm{suf}\,}}(\mathbb {T})$ is connected. Proposition 15 implies that, for any taboo-free string *s* and $n \ge |s|$, $\varGamma ^s_n(\mathbb {T})$ is connected.

Proposition 20 characterizes the connectivity of every $\varGamma _{n + |s| }^s(\mathbb {T})$ for $|s| \ge M$. We know from Theorem 16 that there exists ${r \in {{\,\mathrm{lsc}\,}}(\mathbb {T}) \subseteq {{\,\mathrm{suf}\,}}(V_M(\mathbb {T})) \bigcap {{\,\mathrm{suf}\,}}(\mathbb {T})}$ such that ${\varGamma _{n+|s|}^s(\mathbb {T}) \simeq \varGamma _{n+|r|}^r(\mathbb {T})}$. Since ${{{\,\mathrm{ssc}\,}}(\mathbb {T}) := {{\,\mathrm{suf}\,}}(V_M(\mathbb {T})) \bigcap {{\,\mathrm{suf}\,}}(\mathbb {T}) - {{\,\mathrm{lsc}\,}}(\mathbb {T})}$, to complete our characterization of the connectivity of every taboo-free Hamming graph, some cases (such as Example 6) require considering the connectivity of graphs $\varGamma ^p_n(\mathbb {T})$ for ${p \in {{\,\mathrm{ssc}\,}}(\mathbb {T})}$. We have the following.

### Proposition 21

Given a left proper $\mathbb {T}$ and $p \in {{\,\mathrm{ssc}\,}}(\mathbb {T})$, assume that, for every $r \in {{\,\mathrm{lsc}\,}}(\mathbb {T})$, graph $\varGamma ^r_{M + |r|}(\mathbb {T})$ is connected. Given $k \in \mathbb {N}$, if partition

$$\begin{aligned} V^p_{|p|+k + M-1}(\mathbb {T}) = \bigsqcup _{w \in L^{k}(p)} V^{wp}_{|p|+k+M-1} (\mathbb {T})\end{aligned}$$

satisfies that $(wp)[1, k_{wp}] \in {{\,\mathrm{lsc}\,}}(\mathbb {T})$ for each $w \in L^{k}(p)$, and moreover $\mathcal {Q}[\varGamma ^p_{|p|+k+M-1}(\mathbb {T})]$ is connected, then $\varGamma ^p_n(\mathbb {T})$ is connected for $n \ge |p|+k$.

### Proof

For $n \ge |p|+k$, given partition

$$\begin{aligned} V^p_{n}(\mathbb {T}) = \bigsqcup _{w \in L^{k}(p)} V^{wp}_{n} (\mathbb {T}), \end{aligned}$$

subgraphs $\varGamma ^{wp}_{n} (\mathbb {T})$ are connected due to $(wp)[1, k_{wp}] \in {{\,\mathrm{lsc}\,}}(\mathbb {T})$. Moreover, since $\mathcal{Q}[ \varGamma ^p_{2M-1}(\mathbb {T})]$ is connected, Lemma 18 with $s = p$ implies that $\mathcal{Q}[\varGamma ^p_{n}(\mathbb {T})]$ is connected for $n \ge |p|+k$. Thus, the quotient graph $\mathcal{Q}[\varGamma ^p_{n}(\mathbb {T})]$ and all induced subgraphs $\varGamma ^{wp}_{n} (\mathbb {T})$ are connected. The connectivity of $\varGamma ^p_{n}(\mathbb {T})$ follows applying Proposition 1. $\square$

In Proposition 21, one can always take $k = M-|p|$ and just check if $\mathcal{Q}[ \varGamma ^p_{2M-1}(\mathbb {T})]$ or $\varGamma ^p_{2M-1}(\mathbb {T})$ is connected for $p \in {{\,\mathrm{ssc}\,}}(\mathbb {T})$. Otherwise one can try $k = 1$ and increase it progressively.

### Example 8

If $\varSigma = \{ A, C, G, T\}$ and ${\mathbb {T} = \{ AA,\; CC, \; GG, \;TT \}}$, then it holds that ${{{\,\mathrm{lsc}\,}}(\mathbb {T}) = \{ A, C, G, T\}}$ and ${{{\,\mathrm{ssc}\,}}(\mathbb {T}) = \{ e \} }$. For $r \in {{\,\mathrm{lsc}\,}}(\mathbb {T})$, it can be proven that $\varGamma ^r_{3}(\mathbb {T})$ is connected. Thus, Proposition 20 implies that every $\varGamma ^r_{n}(\mathbb {T})$ is connected for $r \in {{\,\mathrm{lsc}\,}}(\mathbb {T})$ and $n \ge 1$.

We can combine Propositions 20 and 21 to obtain our aimed characterization of the connectivity of every suffix Hamming graph. We do so in the following theorem.

### Theorem 22

Given a left proper taboo-set $\mathbb {T}$, the following are equivalent.

- Consider, for every $r \in {{\,\mathrm{lsc}\,}}(\mathbb {T})$, partition $V^r_{M + |r|}(\mathbb {T}) = \bigsqcup _{a \in L^1(r)} V^{ar}_{M + |r|}(\mathbb {T})$, and for every ${p \in {{\,\mathrm{ssc}\,}}(\mathbb {T})}$, partition $V^p_{2M-1}(\mathbb {T}) = \bigsqcup _{w \in L^{M-|p|}(p)} V^{wp}_{2M-1} (\mathbb {T})$.
  For $r \in {{\,\mathrm{lsc}\,}}(\mathbb {T})$, every partition graph $\mathcal {Q}[\varGamma ^r_{M + |r|}(\mathbb {T})]$ is connected; for $p \in {{\,\mathrm{ssc}\,}}(\mathbb {T})$, every partition graph $\mathcal {Q}[\varGamma ^p_{2M-1}(\mathbb {T})]$ is connected; for $p \in {{\,\mathrm{ssc}\,}}(\mathbb {T})$, every graph $\varGamma ^p_n(\mathbb {T})$ with ${|p| +2 \le n \le M-1}$ is connected.
- For $r \in {{\,\mathrm{lsc}\,}}(\mathbb {T})$, graph $\varGamma ^r_{M + |r|}(\mathbb {T})$ is connected; for ${p \in {{\,\mathrm{ssc}\,}}(\mathbb {T})}$, graph $\varGamma ^p_{2M-1}(\mathbb {T})$ is connected; for ${p \in {{\,\mathrm{ssc}\,}}(\mathbb {T})}$ and ${|p| +2 \le n \le M-1}$, every graph $\varGamma ^p_n(\mathbb {T})$ is connected.
- For every taboo-free string *s* and $n \ge 0$, graph $\varGamma ^s_{|s|+n}(\mathbb {T})$ is connected.

### Proof

Proposition 2 states that the connectivity of a graph is equivalent to the connectivity of each of its quotient graphs. Hence (b) $\Rightarrow$ (a) follows, because if graphs $\varGamma ^r_{M + |r|}(\mathbb {T})$ and $\varGamma ^p_{2M-1}(\mathbb {T})$ are connected, then also partition graphs $\mathcal {Q}[\varGamma ^r_{M + |r|}(\mathbb {T})]$ and $\mathcal {Q}[\varGamma ^p_{2M-1}(\mathbb {T})]$ are connected. Since the implication (c) $\Rightarrow$ (b) is obvious, it only remains to prove (a) $\Rightarrow$ (c).

Theorem 16 states that, when $\mathbb {T}$ is left proper, every nonempty graph of the form $\varGamma _{n+|s|}^s(\mathbb {T})$ is isomorphic to graph $\varGamma _{n+|w|}^w(\mathbb {T})$, where $w = s[1, k_s] \in {{\,\mathrm{suf}\,}}(\mathbb {T}) \bigcap {{\,\mathrm{suf}\,}}(V_M(\mathbb {T}))$. By construction, strings in ${{\,\mathrm{suf}\,}}(\mathbb {T}) \bigcap {{\,\mathrm{suf}\,}}(V_M(\mathbb {T}))$ either belong to ${{\,\mathrm{lsc}\,}}(\mathbb {T})$ or ${{\,\mathrm{ssc}\,}}(\mathbb {T})$. Therefore, statement (c) is equivalent to the connectivity, for every $n \ge 0$, of every $\varGamma ^r_{n+|r|}(\mathbb {T})$, where $r\in {{\,\mathrm{lsc}\,}}(\mathbb {T})$, and of every $\varGamma ^p_{n+|p|}(\mathbb {T})$, where $p\in {{\,\mathrm{ssc}\,}}(\mathbb {T})$.

Assuming statement (a), since every partition graph $\mathcal {Q}[\varGamma ^r_{M + |r|}(\mathbb {T})]$ is connected for $r \in {{\,\mathrm{lsc}\,}}(\mathbb {T})$, Proposition 20 implies that every graph $\varGamma ^w_{M+n}(\mathbb {T})$ is connected, where ${w \in V_M(\mathbb {T})}$ and ${n \ge 0}$. For any ${r \in {{\,\mathrm{lsc}\,}}(\mathbb {T})}$, there exists by construction a ${w \in V_M(\mathbb {T})}$ such that $r = w[1, k_w]$. Since ${\varGamma ^w_{M+n}(\mathbb {T}) \simeq \varGamma ^r_{|r|+n}(\mathbb {T})}$ due to Proposition 15, it follows that (a) implies that every $\varGamma ^r_{|r|+n}(\mathbb {T})$ is connected, where $r\in {{\,\mathrm{lsc}\,}}(\mathbb {T})$ and $n \ge 0$.

It remains to prove that (a) implies that every $\varGamma ^p_{|p|+n}(\mathbb {T})$ is connected, where $p\in {{\,\mathrm{ssc}\,}}(\mathbb {T})$ and $n \ge 0$. Since every partition graph $\mathcal {Q}[\varGamma ^p_{2M-1}(\mathbb {T})]$ is connected, Proposition 21 with $k = M-|p|$ implies that $\varGamma ^p_{M+n}(\mathbb {T})$ is connected for $n \ge 0$. The connectivity of graphs $\varGamma ^p_{|p|+2}(\mathbb {T}), \dots , \varGamma ^p_{M-1}(\mathbb {T})$ is part of the assumptions of (a), and graphs $\varGamma ^p_{|p|+1}(\mathbb {T})$ and $\varGamma ^p_{|p|}(\mathbb {T})$ are trivially connected, finishing the proof. $\square$

In general, if $\mathbb {T}$ has just a few taboos, proving connectivity becomes easier since most of strings are left *k*-synchronized. In Proposition 23 only previous results are used, while in Proposition 24 we study this case more exhaustively in a self-contained manner. Note that, when taboo-set $\mathbb {T}$ is minimal, the assumptions of Proposition 24 are much easier to check.

### Proposition 23

Given a left proper $\mathbb {T}$ such that every pair of strings ${w_1, w_2 \in V_M(\mathbb {T})}$ with $d(w_1, w_2) = 1$ is left 1-synchronized, it holds that:

- For any $r \in {{\,\mathrm{lsc}\,}}(\mathbb {T})$ and $n \in \mathbb {N}_0$, $\varGamma ^r_{n + |r|}(\mathbb {T})$ is connected.
- For any $p \in {{\,\mathrm{ssc}\,}}(\mathbb {T})$ with connected $\varGamma ^p_{M}(\mathbb {T})$, $\varGamma ^p_n(\mathbb {T})$ is connected for $n \ge M$.

### Proof

Proposition 10 implies that every pair $w_1, w_2 \in V_M(\mathbb {T})$ is left *k*-synchronized for any $k \in \mathbb {N}$. We know from Lemma 17 that left *k*-synchronization of two strings with Hamming distance 1 indexing a partition as suffixes is equivalent to those two strings being adjacent in the partition graph. Therefore any quotient graph $\mathcal {Q}[\varGamma ^w_n(\mathbb {T})] = (L^1(w), E_{L^1(w)})$ induced by partition $V^w_{n}(\mathbb {T}) = \bigsqcup _{a \in L^1(w)} V^{aw}_{n}(\mathbb {T})$ is fully connected (that is, every two vertices are adjacent). In particular, every $\mathcal {Q}[\varGamma ^w_n(\mathbb {T})]$ is connected, and thus Proposition 20 implies (a). Similarly with partition $V{^p_{n}(\mathbb {T}) = \bigsqcup _{w \in L^{M-|p|}(p)} V^{wp}_{n} (\mathbb {T}),}$ since ${\mathcal {Q}[\varGamma ^p_n(\mathbb {T})] \simeq \varGamma ^p_{M}(\mathbb {T})}$ for $n \ge M$, Proposition 21 implies (b). $\square$

### Example 9

For $\varSigma = \{ A, C, G, T\}$ and $\mathbb {T} = \{ AA,\; CCC\}$, the strings $Tw_1$ and $Tw_2$ are taboo-free for $w_1, w_2 \in V_3(\mathbb {T})$, hence they are left 1-synchronized. Since ${{\,\mathrm{lsc}\,}}(\mathbb {T}) = {{\,\mathrm{suf}\,}}(\mathbb {T})$, for any taboo-free string *s* and $n \ge |s|$, $\varGamma ^s_n(\mathbb {T})$ is connected.

### Proposition 24

Given taboo-set $\mathbb {T}$ and set $\varPsi (\mathbb {T}) := \bigcup _{t \in \mathbb {T}} t[2, |t|]$, if every pair of taboo-free strings $w_1, w_2 \in \varPsi (\mathbb {T})$ with $|w_1| \ge |w_2|$ and ${d \big ( \; w_1[1, |w_2|]\;, \; w_2 \; \big ) \le 1}$ is left 1-synchronized, then it holds that:

- Every taboo-free string is 1-prefixable. In particular, $\mathbb {T}$ is left proper.
- Every two taboo-free strings $s_1, s_2$ with $d(s_1, s_2) = 1$ are left 1-synchronized.
- Graph $\varGamma ^s_n(\mathbb {T})$ is connected for every taboo-free string *s* and $n \ge |s|$.

### Proof

- Consider any taboo-free string *s*. Assume that, for each $a \in \varSigma$, *as* is not taboo-free, that is, that for some integer $c_a \ge 2$, $(as)[1, c_a] \in \mathbb {T}$. WLOG assume $c_{a_1} \le \cdots \le c_{a_m}$ and consider $s[1, c_{a_m}-1]$, which satisfies $s[1, c_{a_m}-1] \in \varPsi (\mathbb {T})$ since $(a_ms)[1, c_{a_m}] \in \mathbb {T}$. By construction, for any $a \in \varSigma$, string $as[1, c_{a_m}-1]$ is not taboo-free. On the other hand, the Hamming distance between $s[1, c_{a_m}-1] \in \varPsi (\mathbb {T})$ and itself is 0, and thus the assumption of the statement implies that $s[1, c_{a_m}-1]$ is left 1-synchronized with $s[1, c_{a_m}-1]$. In other words, a symbol $a \in \varSigma$ exists such that $as[1, c_{a_m}-1]$ is taboo-free, which is a contradiction. All in all, *s* must be 1-prefixable. Taking $s \in V_M(\mathbb {T})$ we see that $\mathbb {T}$ is left proper.
- Given taboo-free strings $s_1, s_2$ such that $d(s_1, s_2) = 1$, assume that they are not 1-synchronized. Then for every $a \in \varSigma$, either ${(as_1)[1, c_a] \in \mathbb {T}}$ or ${(as_2)[1, c_a] \in \mathbb {T}}$ for some $c_a \ge 2$. Denote by ${C_1 \subseteq \bigcup _{a \in \varSigma }\{ c_a\}}$ those $c_a$ such that ${(as_1)[1, c_a] \in \mathbb {T}}$, and analogously with $C_2$. If $C_1$ were empty, then $s_2$ would not be 1-prefixable, contradicting (a). Thus, both $C_1$ and $C_2$ must be nonempty. Consider ${d_1 := \max \{ c: c \in C_1 \} }$ and ${d_2 := \max \{ c: c \in C_2 \}}$. It holds that ${s_1[2, d_1] \in \varPsi (\mathbb {T})}$ and ${s_2[2, d_2] \in \varPsi (\mathbb {T}) }$. Moreover, we have that the pair ${s_1[2, d_1]}$, ${s_2[2, d_2]}$ is not left 1-synchronized. Since ${d(s_1, s_2) = 1}$, that contradicts the assumptions of the statement, hence $s_1$ and $s_2$ must be left 1-synchronized, as desired.
- Clearly $\varGamma ^s_{|s|}(\mathbb {T})$ is connected, so let us proceed by induction. Assume $\varGamma ^s_n(\mathbb {T})$ is connected for a fixed $n \ge |s|$ and consider $\varGamma ^s_{n+1}(\mathbb {T})$. Since ${V^s_{n+1}(\mathbb {T}) \subseteq \varSigma \circ V^s_n(\mathbb {T})}$, if $|V^s_{n}(\mathbb {T})| = 1$, then $\varGamma ^s_{n+1}(\mathbb {T})$ is connected. Otherwise we take different $s_1, s_2 \in V^s_{n+1}(\mathbb {T})$; we will prove that they are connected. We know that $s_1, s_2 \in \varSigma \circ V^s_n(\mathbb {T})$, hence let us write $s_1 = c_1 w_1$ and $s_2 = c_2 w_2$ for $c_i \in \varSigma$ and $w_i \in V^s_n(\mathbb {T})$. If $w_1 = w_2$, the result is obvious, so assume $w_1 \ne w_2$.
  By hypothesis, $\varGamma ^s_n(\mathbb {T})$ is connected, and thus there exists a path of vertices of $V^s_n(\mathbb {T})$, namely $y_1, \dots , y_D$, such that $d(y_i, y_{i+1}) = 1$, $y_1 = w_1$ and $y_D = w_2$. For every $j \in [1, D-1]$, the pair $y_j$, $y_{j+1}$ is left 1-synchronized, and thus there exists $b_j \in \varSigma$ such that $b_jy_j$ and $b_jy_{j+1}$ are taboo-free. Since $d(b_jy_j, b_jy_{j+1}) = 1$, $b_jy_j$ and $b_jy_{j+1}$ are adjacent in $\varGamma ^s_{n+1}(\mathbb {T})$. Moreover every pair of taboo-free strings contained in $\varSigma \circ y_{i}$ is adjacent for $i \in [1, D-1]$. Since the relation “being connected” is transitive, vertices $s_1 \in \varSigma \circ y_1$ and $s_2 \in \varSigma \circ y_D$ are connected, as desired.$\square$

### Example 10

If $\varSigma = \{ A, C, G, T\}$ and $\mathbb {T} = \{ AA,\; CC,\; GG, \;TT \}$, then $\varPsi (\mathbb {T}) = \{ A, C, G, T\}$. Every pair of strings in $\varPsi (\mathbb {T})$ is left 1-synchronized, hence for every taboo-free *s* and $n \ge |s|$, $\varGamma ^s_n(\mathbb {T})$ is connected.

Now we aim to find an upper bound for the number of taboos needed to guarantee connectivity of the graphs $\varGamma ^s_n(\mathbb {T})$. The following Corollary of Proposition 24 holds.

### Corollary 25

Consider an alphabet $\varSigma$ and a taboo-set $\mathbb {T}$. The following holds:

- If $|\mathbb {T}[1,1]| < |\varSigma |$, then for any taboo-free string *s* and $n \ge |s|$, $\varGamma ^s_{n}(\mathbb {T})$ is connected.
- If $|\mathbb {T}| < |\varSigma |$, then for any taboo-free string *s* and $n \ge |s|$, $\varGamma ^s_{n}(\mathbb {T})$ is connected.

### Proof

- Assume that taboo-free strings $s_1$, $s_2$ satisfy $L^1(s_1)\bigcap L^1(s_2) = \emptyset$. That is, for each $a \in \varSigma$, either $as_1$ or $as_2$ has a taboo as prefix, contradicting $|\mathbb {T}[1,1]| < |\varSigma |$. Therefore every two taboo-free strings are left 1-synchronized, so we can apply Proposition 24.c, implying (a).
- If $|\mathbb {T}| < |\varSigma |$, then $|\mathbb {T}[1,1]| < |\varSigma |$. Thus, statement (a) yields the result.$\square$

Corollary 25.b implies that, if $|\mathbb {T}| < |\varSigma |$, then every $\varGamma ^s_n(\mathbb {T})$ is connected. In Examples 11 and 12, we give examples of taboo-sets over an alphabet with $|\varSigma |=2$ and $|\varSigma |>2$ symbols respectively, such that $|\mathbb {T}| = |\varSigma |$ and at least one suffix graph is disconnected. In this sense, the upper bound $|\mathbb {T}| < |\varSigma |$ that guarantees connectivity for every suffix graph cannot be improved.

### Example 11

If $\varSigma = \{ 0, 1\}$ and $\mathbb {T} = \{ 10, \; 01 \}$, then $\mathbb {T}$ is left proper and $|\mathbb {T}[1,1]| = |\mathbb {T}[2,2]| = 2 = |\varSigma |$. For $n\ge 2$, $V_n(\mathbb {T}) = \{ 0\cdots 0, \; 1\cdots 1\}$, which makes $\varGamma _n(\mathbb {T})$ disconnected. The trivial graphs $\varGamma _n^0(\mathbb {T})$ and $\varGamma _n^1(\mathbb {T})$ are both connected.

### Example 12

For $m \ge 3$, $\varSigma = \{ a_1, \dots , a_m\}$ and the left proper taboo-set

$$\begin{aligned}\mathbb {T} = \{ a_3 a_1, \; a_4 a_1, \; a_5 a_1, \; \dots , \; a_m a_1\} \bigsqcup \; \{ a_1 a_2, \; a_2 a_2\},\end{aligned}$$

we claim that $\varGamma _n^{a_1}(\mathbb {T})$ is disconnected for $n \ge 3$. Indeed,

$$\begin{aligned} V_n^{a_1}(\mathbb {T})&= V_n^{a_1a_1}(\mathbb {T}) \; \bigsqcup \; V_n^{a_2a_1}(\mathbb {T}) = \\&= \Big ( V_n^{a_2a_1a_1}(\mathbb {T}) \bigsqcup V_n^{a_1a_1a_1}(\mathbb {T}) \Big ) \; \bigsqcup \; \left( \bigsqcup _{i \in [3, m]} V_n^{a_ia_2a_1}(\mathbb {T}) \right) , \end{aligned}$$

so take $s \in V_n^{a_2a_1a_1}(\mathbb {T}) \bigsqcup V_n^{a_1a_1a_1}(\mathbb {T})$ and $r \in \bigsqcup _{i \in [3, m]} V_n^{a_ia_2a_1}(\mathbb {T})$. It holds that $d(s, r) \ge 2$, hence we found two disconnected components in graph $\varGamma _n^{a_1}(\mathbb {T})$. This is coherent with $|\mathbb {T}[1,1]| = |\varSigma | = m$.

To generalize this example, for $i \in \mathbb {N}_0$, denote by $s_i := a_1 \overset{i)}{\dots } a_1$ the concatenation of *i* $a_1$’s. The taboo-set

$$\begin{aligned} \mathbb {T}_i = \{ a_3 s_i, a_4 s_i, \dots , a_m s_i\} \; \bigsqcup \; \{ a_1a_2s_{i-1}, a_2a_2s_{i-1} \} \end{aligned}$$

satisfies that graph $\varGamma _n^{s_i}(\mathbb {T}_i)$ is disconnected for $n \ge i+2$.

In this section, we have stated various results regarding the connectivity of every suffix Hamming graph given a left proper taboo-set $\mathbb {T}$. Up to Theorem 16, our aim was to characterize the connectivity of every suffix Hamming graph. Then we found sufficient conditions in Proposition 24 and Corollary 25 that are easier to apply. When studying this connectivity problem, the practitioner should firstly try to apply the results requiring easy-to-check assumptions, and increasingly use the more complicated ones. Given a taboo-set $\mathbb {T}$, a possible workflow would be the following:

1. We check if $|\mathbb {T}[1,1]| < |\varSigma |$. If it holds, we can apply Corollary 25.a. Otherwise go to step 2)
2. In order to apply Proposition 24, we check if every pair of taboo-free strings $w_1, w_2 \in \varPsi (\mathbb {T})$ with $|w_1| \ge |w_2|$ and ${d \big ( \; w_1[1, |w_2|]\;, \; w_2 \; \big ) \le 1}$ is left 1-synchronized. If it does not hold, go to step 3)
3. We check whether $\mathbb {T}$ is left proper (this holds in all the biological examples that we considered so far). Otherwise redefine an equivalent left proper taboo-set and apply the characterization of Theorem 22. Two possibilities can arise: Either every suffix Hamming graph is connected, and thus evolution can explore all the space of taboo-free strings; or some taboo-free strings belonging to ${{\,\mathrm{lsc}\,}}(\mathbb {T})$ or ${{\,\mathrm{ssc}\,}}(\mathbb {T})$ induce disconnected suffix graphs $\varGamma ^s_{n_0}(\mathbb {T})$ for some $n_0 \ge |s| + M$, implying that $\varGamma ^s_{n}(\mathbb {T})$ stays disconnected for $n \ge n_0$.

## Examples of plausible bacterial taboo-sets

Taboo-sets as generated by the avoidance of restriction sites can assume various levels of complexities. In this section, we discuss some examples from REBASE (Roberts et al. 2014) using the theory developed in this work. Note that many restriction enzymes of REBASE database have an unknown recognition site, hence our taboo-sets may underestimate the actual amount of taboos. Before describing the examples, we will briefly review essential nomenclature for DNA sequences.

DNA is double-stranded, where *A* pairs with *T* and *G* pairs with *C*, hence it suffices to discuss only one of the strands. We adopt the convention that, given any of the strands, the DNA sequence is always represented from the 5’ end to the 3’ end (which is chemically determined). As a consequence, given a DNA sequence, **its complementary DNA sequence**, the one lying on the opposite strand, is obtained by inverting the order of the symbols and carrying through substitutions $A \leftrightarrow T$ and $C \leftrightarrow G$. If a DNA sequence *s* is identical to its complementary DNA sequence, we say that *s* is an **inverted repeat** (Ussery et al. 2008). For example, sequence *CCGG* is an inverted repeat.

The fact that DNA is double-stranded implies that each recognition site induces taboos in pairs, namely itself and its complementary DNA sequence. For example, if *AGGGC* is a recognition site, then also the complementary strand *GCCCT* is a taboo. If, however, the recognition site is an inverted repeat such as *TGCA*, then this pair is actually one single recognition site. Recognition sites of type II R–M systems are nearly always an inverted repeat (Rusinov et al. 2015; Gelfand and Koonin 1997), and therefore one recognition site induces one single taboo. This is specially interesting because, according to Rusinov et al. (2015, (2018a), only type II R–M systems induce taboos.

A permutation of the symbols of alphabet $\varSigma$ does not alter any of the results that we proved along this work. Moreover, by reversing the order of the symbols, any statement regarding e.g. left-properness and suffixes has an analogous one in which right-properness and suffixes are involved. On the other hand, taboo-sets induced by restriction enzymes remain invariant when we interchange every recognition site by its complementary sequence. Therefore, note that, for a bacterial taboo-set $\mathbb {T}$, if we prove that every graph $\varGamma ^s_n(\mathbb {T})$ is connected, then also every graph $^s\varGamma _n(\mathbb {T})$ is connected.

### A frequent case: *Turneriella parva*

The * Turneriella parva * (REBASE organism number 8970) strain produces a restriction enzyme with recognition site *GATC*, an inverted repeat. Similarly, another of its enzymes has recognition sites *GGACC* and *GGTCC*. Thus, these restriction enzymes generate the taboo-set

4 $$\begin{aligned} \mathbb {T}_{T.pa} = \{ GATC \} \bigcup \{ GGACC, GGTCC \}. \end{aligned}$$

Since $|\mathbb {T}_{T.pa}[1,1]| < 4$, Corollary 25.a implies that every graph $\varGamma _n^s( \mathbb {T}_{T.pa})$ is connected. Therefore the evolution of the DNA sequences can potentially reach any other taboo-free DNA sequence, no matter which suffix was conserved along this process.

Among the 3623 bacteria in REBASE (2020a), only 465 have more than three type II restriction enzymes. Assuming that only type II restriction enzymes induce taboos, as stated by Rusinov et al. (2015, (2018a), Corollary 25.b implies that at least $87\% \; (3158/3623)$ of bacterial taboo-sets in REBASE (2020a) yield connected taboo-free Hamming graphs. Similarly, at least $90 \% \; (139/153)$ of archea in REBASE (2020b) induce connected taboo-free Hamming graphs, because they have less than four type II restriction enzymes. The following example describes a more complex collection of restriction enzymes.

### *Helicobacter pylori*

In *H. pylori* 21-A-EK1, studied by Ailloud et al. (2019), many restriction enzymes have been identified. For the sake of clarity, let us write ${\mathbb {T}_{H.py} = {^A\mathbb {T}} \bigcup {^G\mathbb {T}} \bigcup {^C\mathbb {T}} \bigcup {^T\mathbb {T}}}$, where ${^a\mathbb {T}}$ denotes those taboos in $\mathbb {T}_{H.py}$ whose **first** symbol is $a \in \varSigma$. Then we have

5 $$\begin{aligned} \begin{aligned} {^A\mathbb {T}}&= \{ AC \circ \varSigma \circ GT \},\\ {^G\mathbb {T}}&= (GT \circ \varSigma ^2 \circ AC) \bigcup \{ GTCAC, GTGAC\}\\&\quad \bigcup \{ GTAC, GAGG\}\\ {^C\mathbb {T}}&= \{ CCGG, CCTC, CATG\},\\ {^T\mathbb {T}}&= \{TGCA\},\\ \end{aligned} \end{aligned}$$

where $GT \circ \varSigma ^2 \circ AC$ represents taboos of the type *GTabAC* with $a,b \in \varSigma$, and so on for analogous notations.

We want to apply Proposition 24. Take any $r_1, r_2 \in \varPsi (\mathbb {T}_{H.py})$ and assume that they are **not** left 1-synchronized. In particular WLOG we can assume that ${T \notin L^1(r_1)}$, implying ${r_1 = GCA}$. If ${C \notin L^1(r_1)}$, then ${r_1 \in \{ CGG, CTC, ATG\}}$, which contradicts ${r_1 = GCA}$. Therefore it must be ${C \notin L^1(r_2)}$, yielding $r_2 \in \{ CGG, CTC, ATG\}$. In any case, $d(r_1, r_2) \ge 2$. Thus, for any $w_1, w_2 \in \varPsi (\mathbb {T})$ with ${d \big ( \; w_1[1, |w_2|]\;, \; w_2 \; \big ) \le 1}$, it holds that $w_1$ and $w_2$ are left 1-synchronized, so Proposition 24 can be applied: Every graph $\varGamma ^s_n(\mathbb {T}_{H.py})$ is connected and, in particular, $\varGamma _n(\mathbb {T}_{H.py})$ is connected.

### An imaginary bacterium

The taboo-set can significantly influence evolution in the cases where some $\varGamma ^s_n(\mathbb {T})$ is disconnected. To explain this, we will create a plausible, nonexistent example. Suppose that a strain of *Bacterium imaginara* has taboo-set

$$\begin{aligned} \mathbb {T}_{B.im} = \{ ACCC, TCCC, CGCC, GGCC \} \bigcup \{ GGGT, GGGA, GGCG\},\end{aligned}$$

where the second set contains the complementary DNA sequences of the first set, except that of *GGCC*, which is an inverted repeat. Thus, taboo-set $\mathbb {T}_{B.im}$ is induced by 4 restriction enzymes. At first glance, taboo-set $\mathbb {T}_{B.im}$ seems less restrictive than $\mathbb {T}_{H.py}$, which has 6 taboos of length four and 22 taboos of length five or more.

Proposition 24 cannot be applied because *CCC* and *GCC* are not left 1-synchronized, and actually we can find a disconnected suffix graph. Let us take $V^{CCC}_n(\mathbb {T}_{B.im})$, which satisfies

$$\begin{aligned}&V^{CCC}_n(\mathbb {T}_{B.im}) = V^{GCCC}_n(\mathbb {T}_{B.im}) \bigcup \; V^{CCCC}_n(\mathbb {T}_{B.im}) \\&\quad = \Big ( V^{AGCCC}_n(\mathbb {T}_{B.im}) \bigcup V^{TGCCC}_n(\mathbb {T}_{B.im}) \Big ) \bigcup \Big ( V^{GCCCC}_n(\mathbb {T}_{B.im}) \bigcup V^{CCCCC}_n(\mathbb {T}_{B.im}) \Big ), \end{aligned}$$

implying that, for any strings $s_1\in V^{GCCC}_n(\mathbb {T}_{B.im})$ and $s_2 \in V^{CCCC}_n(\mathbb {T}_{B.im})$, it holds that $d(s_1, s_2) \ge 2$. Thus, we found two disconnected components in $\varGamma ^{CCC}_n(\mathbb {T}_{B.im})$, namely $\varGamma ^{GCCC}_n(\mathbb {T}_{B.im})$ and $\varGamma ^{CCCC}_n(\mathbb {T}_{B.im})$. All in all, the graph $\varGamma ^{CCC}_n(\mathbb {T}_{B.im})$ is disconnected for $n \ge 5$.

This produces the following evolutionary implications: Assume that we have two correctly aligned DNA fragments $f_\alpha$ and $f_\beta$ of the genome of *Bacterium imaginara*. Assume moreover that we can write ${f_\alpha = r_{\alpha }GCCC}$ and ${f_\beta = r_{\beta }CCCC}$ for some strings $r_{\alpha }$ and $r_{\beta }$, as also that the suffix *CCC* is invariable due to functional constrains. Then $f_\alpha$ cannot have evolved from $f_\beta$ by simple point mutations, because at some point in evolution a taboo string is produced that is lethal for the carrier. Thus, the standard models of sequence evolution (Strimmer and von Haeseler 2009) do not apply.

## Concluding remarks

Using the results proven in this work, it is possible to decide whether every Hamming graph $\varGamma _n^s(\mathbb {T})$ is connected. The connectivity of the taboo-free Hamming graphs induced by the restriction enzymes of the bacteria listed in REBASE could be quickly analysed with our tools. Unfortunately, for many organisms listed in REBASE, the recognition sites of restriction enzymes are not available.

Based on the current version of REBASE (2020a), we conclude using Corollary 25 that taboo-sets of at least $87\% \; (3158/3623)$ of bacteria in REBASE induce connected taboo-free Hamming graphs, because they have less than four type II restriction enzymes. For larger taboo-sets, Proposition 24 can be used, as we did in Sect. 9.2, or one can directly use the characterization of Theorem 22. Thus, restriction enzymes in bacteria generally do not lead to any disconnected taboo-free Hamming graph, and our models of sequence evolution are by and large applicable. However, the influence of some missing sequences in the Hamming graph on the estimation of evolutionary parameters deserves further investigations. We also would like to emphasize that still many recognition sites have to be identified, and thus it may be well possible that we find disconnected taboo-free Hamming graphs in the next future.

We consider the formal framework developed in this paper as a first and necessary step to understand the effect of restriction enzymes (and possibly other taboo sequences) on the DNA composition of bacteria and viruses, or more generally on the sequence space modelled as a Hamming graph. Consider, for example, the phylogenetic studies by Ailloud et al. (2019), where the *H. pylori* taboo-set $\mathbb {T}_{H.py}$ of Sect.  9.2 was taken from. The following natural questions arise: How are inferred evolutionary times between the two *H. pylori* populations affected by $\mathbb {T}_{H.py}$? Has their *GC* content varied due to the taboos of restriction enzymes?

To answer such questions, we need to develop models of sequence evolution that take taboos into account. Taboo avoidance induces complex dependencies along a DNA sequence, which can be measured using Markov Chain Monte Carlo (MCMC) simulations. If all taboo-free Hamming graphs $\varGamma ^s_n(\mathbb {T})$ are connected, then MCMC methods are easy to apply (Manuel et al. unpublished). A disconnected taboo-free Hamming graph, however, leads to a reducible Markov chain, which complicates simulation of taboo-free evolution.

Another application of our framework is the construction of combinations of restriction enzymes that lead to a disconnected Hamming graph, and thus limit evolutionary freedom. This may help to efficiently treat viral infections. Some progress has been made in the usage of restriction enzymes for the treatment of viral infections (Weber et al. 2014). Since one or just a few SNPs can significantly alter the symptoms or even the mortality associated to a pathogen (Collery et al. 2017; Yuan et al. 2017), our characterization of the connectivity of taboo-free Hamming graphs could help to delete SNPs from the viral genome that are detrimental to humans. Although the treatment of an infection using restriction enzymes is mostly unexplored, this work could be a first theoretical guide to a successful treatment.
